# Mechanistic Insights into Rare Earth Element Dissolution from Sart (Manisa) Black Sand via Alkali Roasting–Acid Leaching

**DOI:** 10.1021/acsomega.6c02633

**Published:** 2026-06-25

**Authors:** Fatih Turan

**Affiliations:** Department of Mining Engineering, 37508 Dokuz Eylül University, Buca, 35210 Izmir, Turkiye

## Abstract

The recovery of rare earth elements (REEs) from REE-bearing heavy mineral tailings of the Manisa–Sart (Türkiye) placer deposit—with an REE-bearing phase consistent with a monazite-group phosphate host, supported by SEM–EDS microanalysis and bulk geochemical evidence—was investigated via an integrated alkali roasting–reductive acid leaching route. The effects of roasting temperature, NaOH stoichiometry, acid type and concentration, reducing agent addition, and the heating method (conventional vs microwave) on REE extraction efficiency were systematically evaluated. Roasting at 600 °C with a 1:1.5 ore-to-NaOH ratio (w/w) provided the most effective structural activation of the REE-bearing matrix, while 4 M hydrochloric acid was identified as the most effective leaching medium; sulfuric acid yielded substantially lower recoveries due to double-sulfate precipitation. The addition of hydrogen peroxide (H_2_O_2_) enhanced cerium extraction approximately 4-fold by reductively converting insoluble Ce­(IV) oxide to soluble Ce­(III) species. Under optimized conditions (4 M HCl + H_2_O_2_, 90 °C, 120 min), neodymium (Nd) and cerium (Ce) extraction efficiencies reached 90.7% and 50.0%, respectively. Dissolution kinetics were analyzed using the Shrinking Core Model (SCM) over a temperature range of 60–90 °C. Both the surface chemical reaction and product layer diffusion control models provided statistically comparable fits (*R* ^2^ ≈ 0.944–0.958), and Arrhenius analysis yielded apparent activation energies of 25.79 and 42.95 kJ/mol, respectively, indicating a surface reaction-dominant mixed-control dissolution mechanism. Microwave-assisted roasting (30 min) achieved a Nd extraction of 80.1%, exceeding conventional furnace roasting (2 h) by 5.3 percentage points. This improvement is consistent with an enhanced structural activation under microwave-assisted heating. Titanium dissolution was limited to 31.5% under all conditions, suggesting a natural process selectivity against titanium under the investigated conditions. These findings provide a mechanistic framework and establish energy-conscious operational parameters for the recovery of REEs from secondary placer resources.

## Graphical abstract

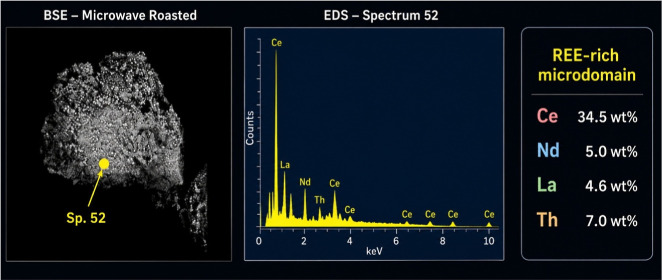

## 1 Introduction

Rare earth elements (REEs) are indispensable to modern high-technology systems due to their unique magnetic, optical, and catalytic properties.^1–3^ The accelerating deployment of renewable energy technologies, electric mobility, and advanced digital infrastructure has significantly increased the demand for critical REEs such as neodymium (Nd), dysprosium (Dy), praseodymium (Pr), and cerium (Ce).^4^ However, the global supply of primary REE resources is geographically concentrated, and their conventional extraction and processing routes are associated with considerable environmental burdens.^5^

These factors have intensified interest in alternative and secondary resources capable of supporting a more resilient and sustainable REE supply chain.^6,21–23,25^ Among secondary resources, placer-type heavy mineral deposits represent polymetallic accumulations formed through natural weathering, transport, and hydraulic sorting processes.^7^ These deposits typically contain mineral assemblages such as zircon, rutile, ilmenite, monazite, and xenotime. While coastal black sands in regions such as Egypt, India, Australia, and Brazil have been extensively studied,^18,49^ the heavy mineral placers of western Anatolia remain comparatively underexplored from a hydrometallurgical perspective.^33^ The Manisa–Salihli (Sart) region, historically known for gold mineralization, hosts heavy mineral tailings enriched in monazite and zircon. Monazite ((Ce, La, Th)­PO_4_) is a significant source of light rare earth elements (LREEs); however, its highly stable phosphate matrix renders it refractory to direct acid leaching.^8,44^ Industrial processing of monazite commonly involves either sulfuric acid “acid baking” or alkali roasting routes.

Acid baking requires elevated temperatures and may generate corrosive SO_*x*_ emissions, posing environmental and operational challenges.^9,27,34^ In contrast, alkali roasting with sodium hydroxide (NaOH) decomposes the phosphate lattice by forming water-soluble sodium phosphate (Na_3_PO_4_) and converting REEs into acid-soluble hydroxides.^10,44^ Despite this structural activation, the efficiency of the subsequent acid leaching stage is often limited by the oxidation of cerium. During roasting, trivalent cerium (Ce^3+^) can be oxidized to tetravalent cerium oxide (CeO_2_), which exhibits very low solubility in conventional mineral acids.^44^ As a result, the overall REE recovery may be substantially reduced.

To address this limitation, reductive leaching strategies have been proposed. The incorporation of hydrogen peroxide (H_2_O_2_) into hydrochloric acid media enables the reduction of Ce^4+^ to soluble Ce^3+^ species, thereby enhancing cerium dissolution and improving the total REE extraction efficiency.^10,16,29^ In parallel, efforts to improve the process sustainability have emphasized energy-efficient thermal treatments. Conventional furnace roasting relies on conductive heat transfer from the particle surface inward, often requiring prolonged residence times.^11^ Microwave-assisted heating, by contrast, enables volumetric and selective energy absorption, which can promote rapid phase transformation and microstructural modification.^28,35^ Such structural changes may influence both intrinsic reaction rates and mass transfer characteristics during leaching.^15^

Although alkali roasting–acid leaching systems have been widely investigated for primary REE ores, systematic kinetic evaluations of thermally pretreated secondary REE-bearing placer tailings remain limited.^17,19^ In particular, the mechanistic transition between surface chemical reaction control and product-layer diffusion control following high-temperature phase transformation has not been quantitatively clarified for secondary placer-derived materials.^49^ A rigorous kinetic assessment is essential to establish the rate-limiting steps and to guide process scale-up.^24,46^

Accordingly, this study aims to (i) optimize the roasting temperature for effective structural activation of the REE-bearing Sart tailings, (ii) compare conventional and microwave-assisted alkali roasting in terms of extraction performance and microstructural evolution, (iii) establish kinetic parameters under controlled H_2_O_2_-assisted reductive leaching conditions, and (iv) elucidate the governing rate mechanism through comparative application of the shrinking core model and Arrhenius analysis. By integrating mineralogical characterization, process intensification, and quantitative kinetic modeling, this work provides a mechanistic framework for energy-conscious and scalable recovery of REEs from secondary placer resources.

## 2 Materials and Methods

The feedstock utilized in this investigation was sourced from the placer deposits of the Salihli–Sart region in Manisa, Türkiye.^7,40^ This region is historically and contemporarily renowned for its intensive gold (Au) mining operations. It should be noted that the utilized sample is not a pristine, as-mined sand; rather, it constitutes the “heavy mineral tailings” generated as a byproduct of gravimetric beneficiation processes (e.g., shaking tables and sluice boxes) employed for primary gold recovery. Bulk chemical analysis indicates that the material is dominated by iron and titanium oxide phases (Fe_2_O_3_: 33.56 wt %; TiO_2_: 21.22 wt %), with rare earth elements and zircon present as trace-to-minor constituents within this complex matrix. Although currently stockpiled in tailing facilities, this Fe–Ti-rich discard material contains REE-bearing phosphate phases—most notably monazite—that may warrant investigation as a secondary resource within a broader circular economy framework.^10,15,32^

### 2.1 Material Supply and Mineralogical Characterization

The placer-type black sand sample utilized in this study was derived from the fine sand to silt size fraction. The quantitative mineralogical composition of the feedstock was elucidated via X-ray diffraction (XRD) analysis coupled to Rietveld refinement. The acquired diffractogram is depicted in Figure 1, while the corresponding phase quantification and chemical composition results are summarized in Tables 1 and 2, respectively.^36–39,42,43^

**Figure 1:**
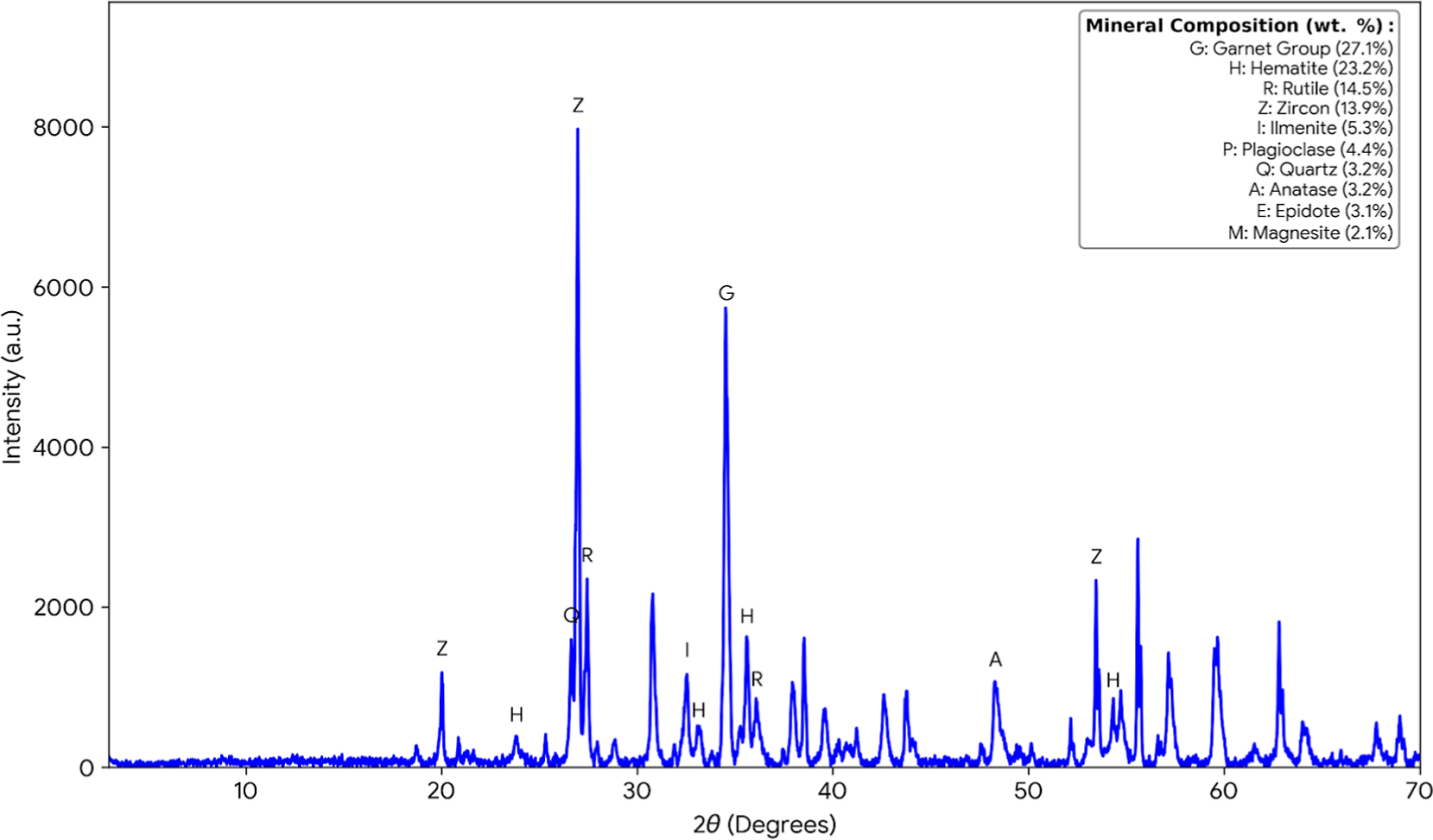
XRD pattern of the Sart heavy mineral tailings showing dominant phases of garnet, hematite, rutile, and zircon. No diffraction peaks attributable to monazite are discernible; this is consistent with trace-level content below the XRD detection threshold and/or radiation-induced metamict amorphization. Monazite presence is inferred from bulk geochemical evidence rather than quantified by Rietveld refinement.

**Table 1:** Mineral Composition and Relative Content of Black Sand

Mineral/Phase Name	Content (wt %)	Mineral/Phase Name	Content (wt %)
Garnet Group (Almandine/Pyrope)	27.1	Plagioclase Feldspar	4.4
Hematite	23.2	Quartz	3.2
Rutile	14.5	Anatase	3.2
Zircon	13.9	Epidote group	3.1
Ilmenite	5.3	Magnesite	2.1
**Total**			**100.0**

**Table 2:** Chemical Composition of Black Sand (Major Oxides, wt %)^a^

Al_2_O_3_	BaO	CaO	Cr_2_O_3_	Fe_2_O_3_	K_2_O	MgO	MnO	Na_2_O	P_2_O_5_	SiO_2_	SO_3_	SrO	TiO_2_	LOI1000
8.54	0.16	5.21	0.08	33.56	0.35	0.81	1.42	0.13	1.07	17.77	0.17	<0.01	21.22	–0.66

a Trace and rare earth element concentrations are presented separately in Table 3.

The XRD data reveal that the bulk matrix is predominantly composed of heavy minerals, specifically the garnet group (27.1%), hematite (23.2%), rutile (14.5%), and zircon (13.9%).^12,40^ Furthermore, minor quantities of ilmenite, plagioclase, quartz, anatase, and epidote were identified as associated gangue minerals. Notably, no discernible diffraction peaks attributable to monazite were detected in the XRD pattern, consistent with its trace-level abundance and probable metamorphic amorphization. The presence of a monazite-group phase as a probable REE carrier is inferred from bulk geochemical evidence, as detailed below,^1,14^ though direct mineralogical confirmation is not available from XRD alone.

Trace and rare-earth element concentrations in the feedstock (ICP–MS, mg/kg) are presented in Table 3. The sample is characterized by a dominant LREE signature, with Ce (1301.8), La (624.1), and Nd (583.34) mg/kg collectively accounting for 81% of total quantified REEs, accompanied by elevated Th (242.03) and Y (378.63) mg/kg.^39^

**Table 3:** Rare Earth Element and Associated Trace Element Concentrations in the Sart Black Sand Feedstock Determined by ICP–MS (mg/kg, i.e., ppm by Mass)^a^

Element	Conc. (mg/kg)	Element	Conc. (mg/kg)	Element	Conc. (mg/kg)
La	624.1	Gd	98.11	Th	242.03
Ce	1301.8	Dy	68.99	U	29.31
Nd	583.34	Er	38.18	Sc	67.77
Pr	155.43	Yb	33.82	Y	378.63
Sm	114.98	Total REE	3207.26	LREE %	81.0%

a Total REE = sum of lanthanides + Y. LREE fraction = La + Ce + Nd + Pr + Sm. Total REE = La + Ce + Nd + Pr + Sm + Gd + Dy + Er + Yb + Y. LREE % = (La + Ce + Nd + Pr + Sm)/total REE × 100.

The detection and elucidation of the mineralogical behavior of monazite, the principal target phase of this study, are supported by multiple lines of geochemical evidence beyond the X-ray diffraction (XRD) pattern. In the XRD diffractogram presented in Figure 1, the characteristic peaks of monazite were not prominently observed. Two primary factors account for this phenomenon. First, the monazite phase is present at trace levels in the bulk matrix, causing its weak diffraction intensities to be effectively masked by the dominant crystalline minerals (i.e., garnet, rutile, and hematite). Second, and more critically from a hydrometallurgical perspective, is the occurrence of “metamictization.” As reported in the literature, monazite can undergo progressive crystal lattice damage over geological time due to the alpha radiation emitted by the radioactive decay of constituent thorium (Th) and uranium (U) isotopes. This radiation-induced structural degradation transforms the mineral into an amorphous (disordered) state, consequently leading to the attenuation or complete disappearance of its characteristic XRD peaks.^37,42,45^

Despite the analytical limitations of XRD, the presence of an REE-bearing phase consistent with a monazite group host is supported by SEM–EDS point analysis of the microwave-roasted calcine together with the bulk geochemical signature of the feedstock (Table 2). The co-occurrence of a pronounced LREE dominance (La, Ce, and Nd accounting for 81.00% of total REEs) and elevated P_2_O_5_ (1.07 wt %) and Th (242.03 ppm) is geochemically consistent with the presence of a monazite-group phosphate [(Ce,La,Nd,Th)­PO_4_] as a principal REE-bearing phase.^13,47^ Critically, SEM–EDS point analysis of the microwave-roasted calcine (Section 3.5 and Figure 15) detected Ce, La, Nd, and Th signals simultaneously within a single microdomain, providing direct microanalytical support for the presence of an REE-bearing phase consistent with the inferred monazite-group host. The absence of detectable REE signals in the raw black sand feedstock (BM sample) is consistent with the submicrometer grain size and metamict structural state of the monazite-group phase, which disperses the REE signal below EDS detection limits at the analytical scale employed. Following microwave-assisted roasting, structural activation and surface exposure of the REE-bearing phase rendered it directly detectable by EDS, providing strong microanalytical support for the monazite-group assignment. Furthermore, it is postulated that this inferred amorphized structure, disrupted by potential metamictization, may exhibit heightened susceptibility to chemical reagents (e.g., during alkali roasting) compared to its highly crystalline counterpart. This structural disorder may confer a potential thermodynamic and kinetic benefit for the subsequent extraction of lanthanides, although this remains to be quantitatively confirmed.^24,45^

**Figure 2:**
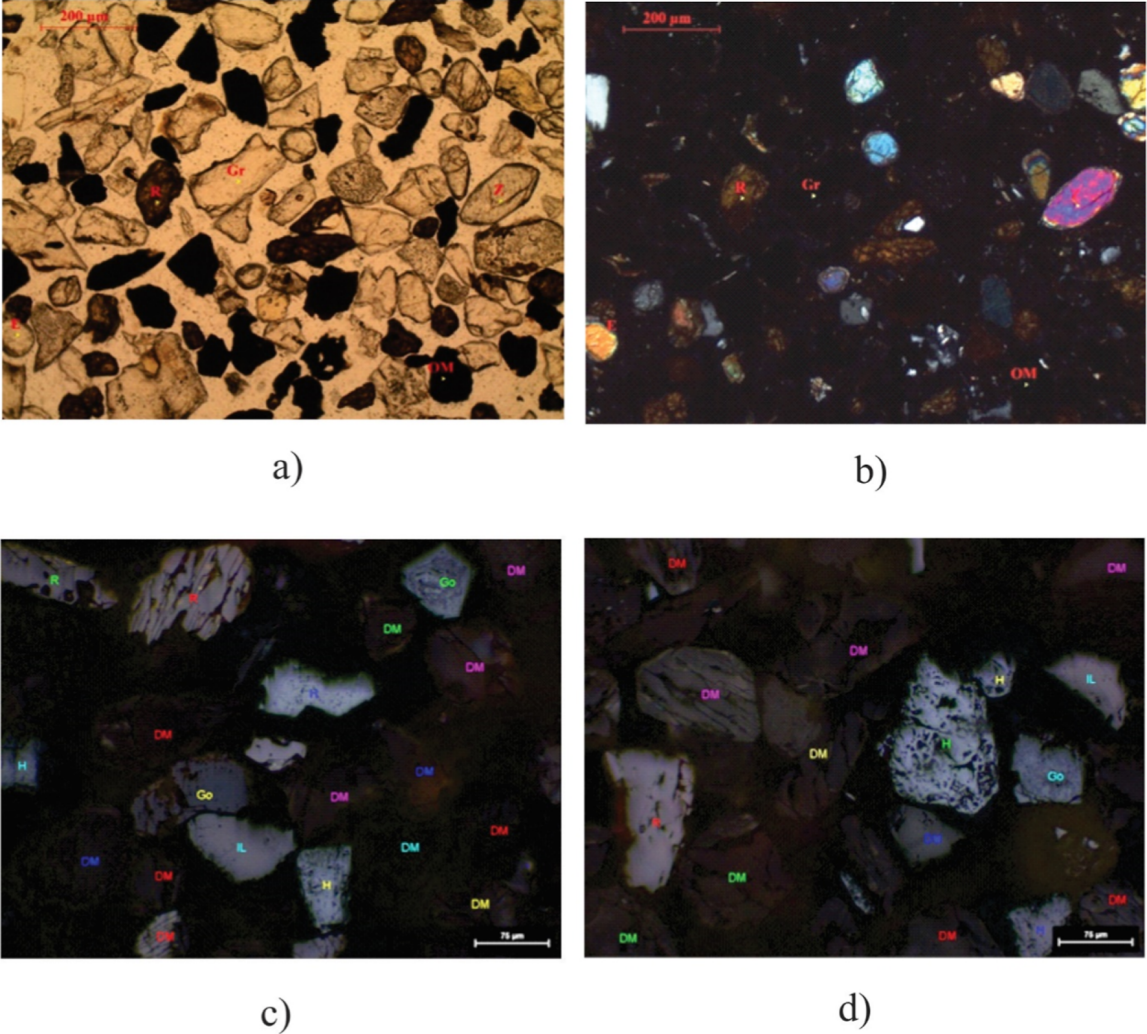
Photomicrographs of the heavy mineral placer sample: (a) general view of the thin section under cross-polarized light (XPL); (b) general view of the thin section under plane-polarized light (PPL); and (c,d) representative views of the polished section under reflected-light ore microscopy, illustrating the opaque mineral assemblages. Photographs taken by the author (F.T.) at the Mineralogy Laboratory, Department of Mining Engineering, Dokuz Eylül University.

**Figure 3:**
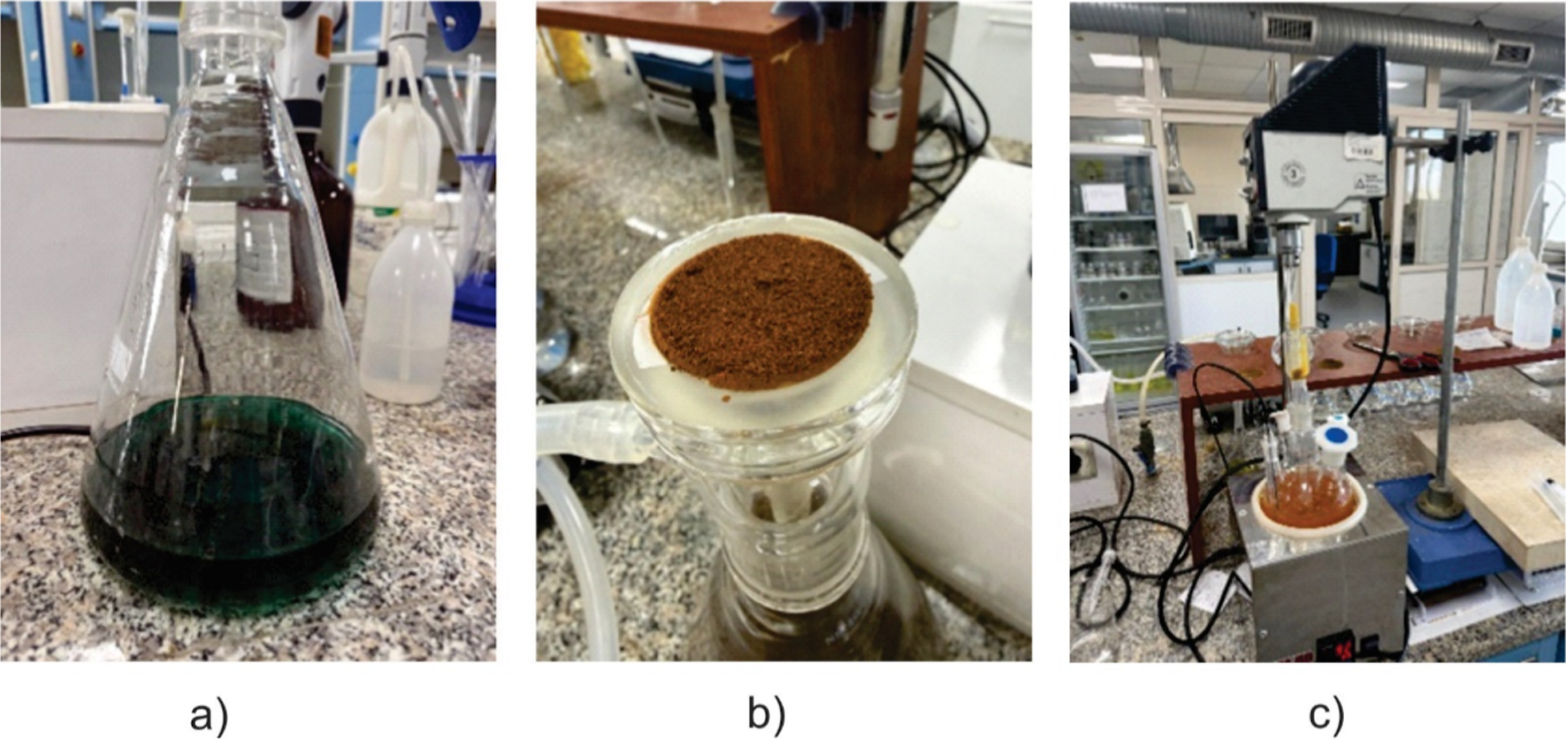
Visual progression of the hydrometallurgical stages: (a) the characteristic green sodium manganate filtrate, indicating the removal of manganese (Mn) and phosphate (P) during the water leaching step; (b) the purified solid residue (calcine) serving as the prepared feedstock for the subsequent acid leaching; and (c) the distinctive orange-colored pregnant leach solution resulting from the dissolution of Fe^3+^ ions during acid leaching. Photographs taken by the corresponding author in the Hydrometallurgy Laboratory, Department of Mining Engineering, Dokuz Eylül University.

**Figure 4:**
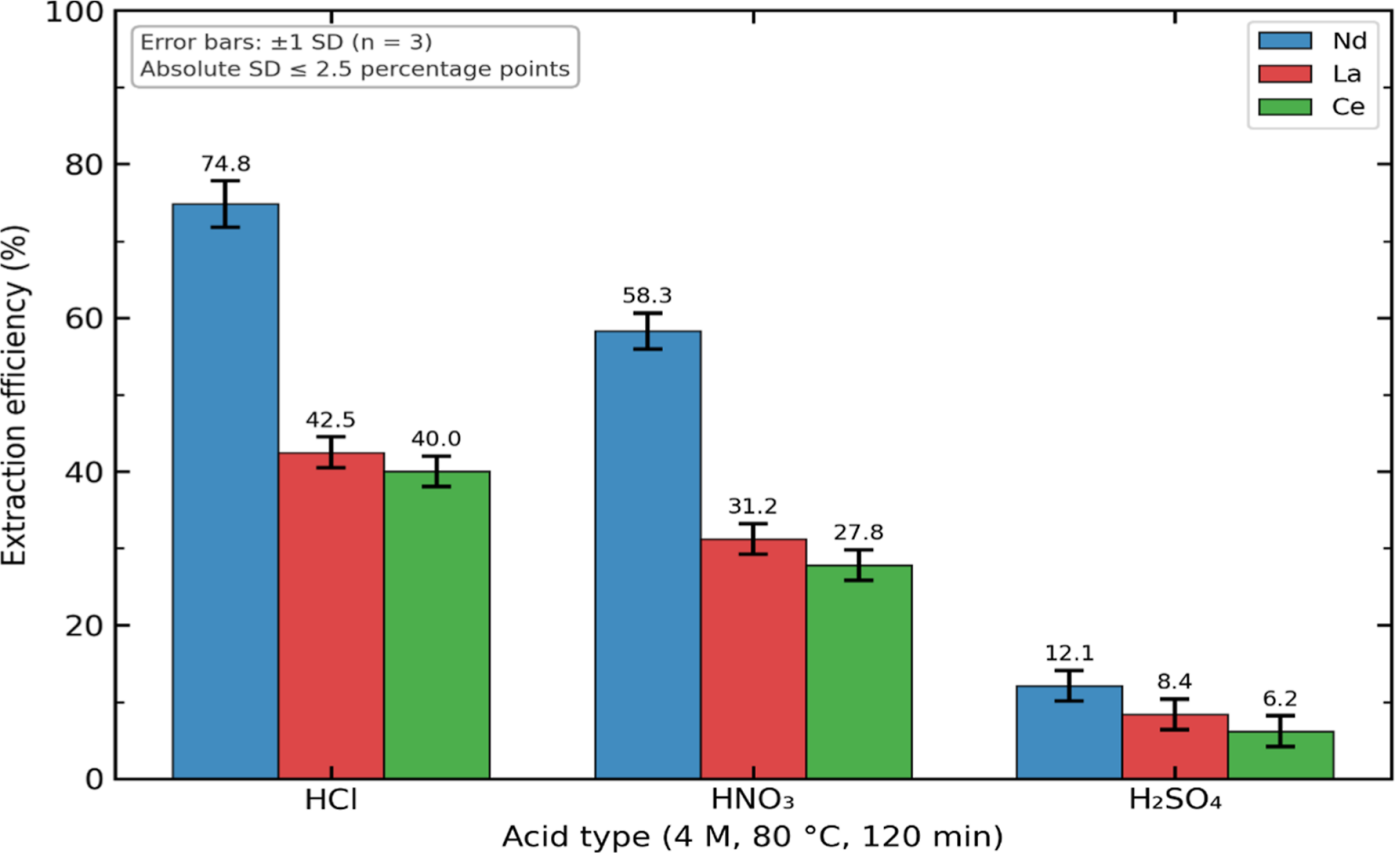
Extraction efficiencies of Nd, La, and Ce as a function of acid type (4 M HCl, HNO_3_, and H_2_SO_4_) following alkali roasting at 600 °C (80 °C, 120 min, +H_2_O_2_). HCl provided the most favorable leaching environment, whereas H_2_SO_4_ resulted in severe recovery losses attributed to double-sulfate coprecipitation. Error bars represent ±1 SD (*n* = 3); absolute SD ≤ 2.5 percentage points.

**Figure 5:**
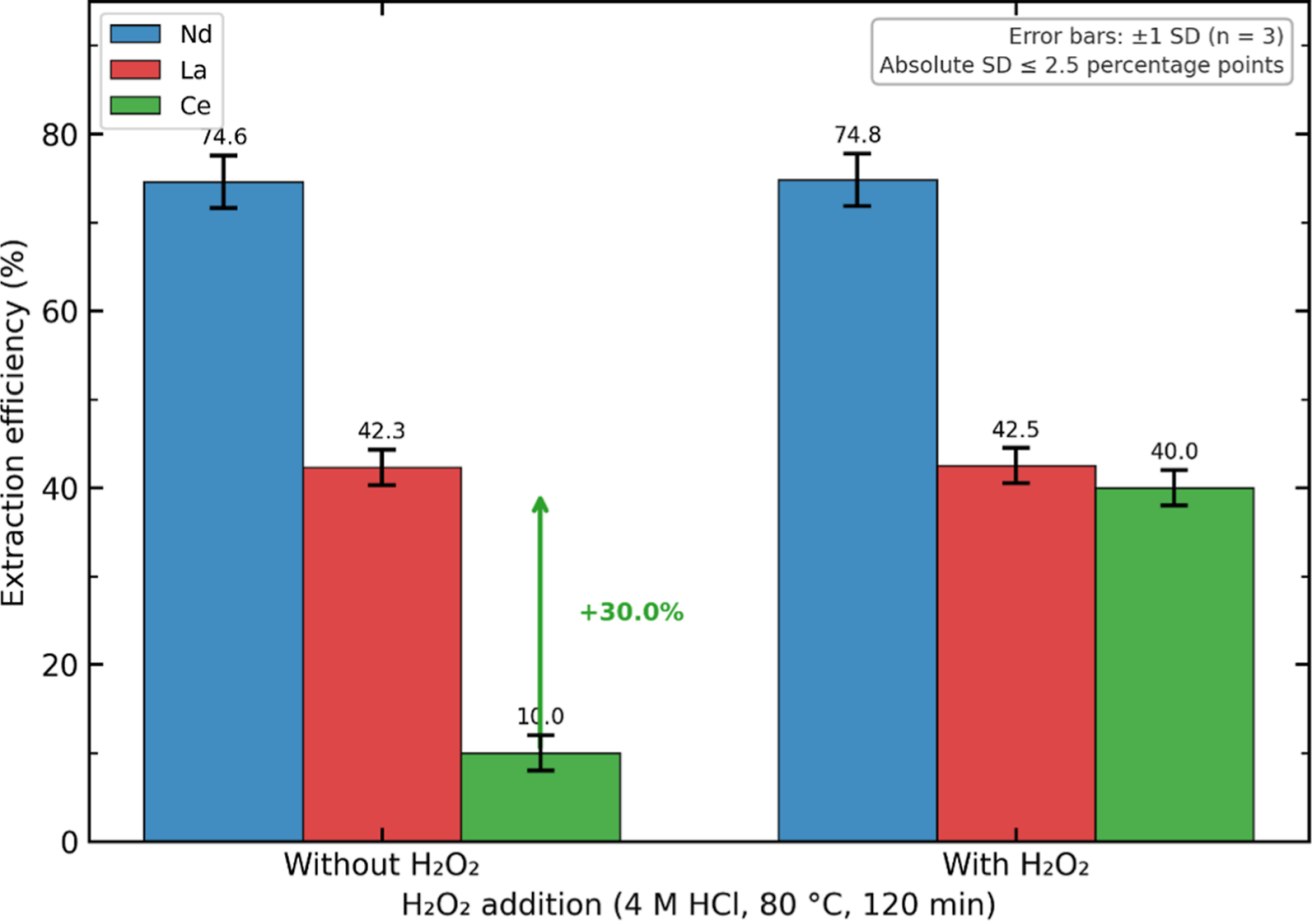
Comparative extraction efficiencies of Nd, La, and Ce with and without H_2_O_2_ addition (4 M HCl, 80 °C, 120 min). The selective enhancement of Ce extraction efficiency by H_2_O_2_ is attributed to reductive conversion of Ce­(IV) oxide to soluble Ce­(III) species, while Nd and La dissolution remained independent of the reductant. Error bars represent ±1 SD (*n* = 3); absolute SD ≤ 2.5 percentage points.

**Figure 6:**
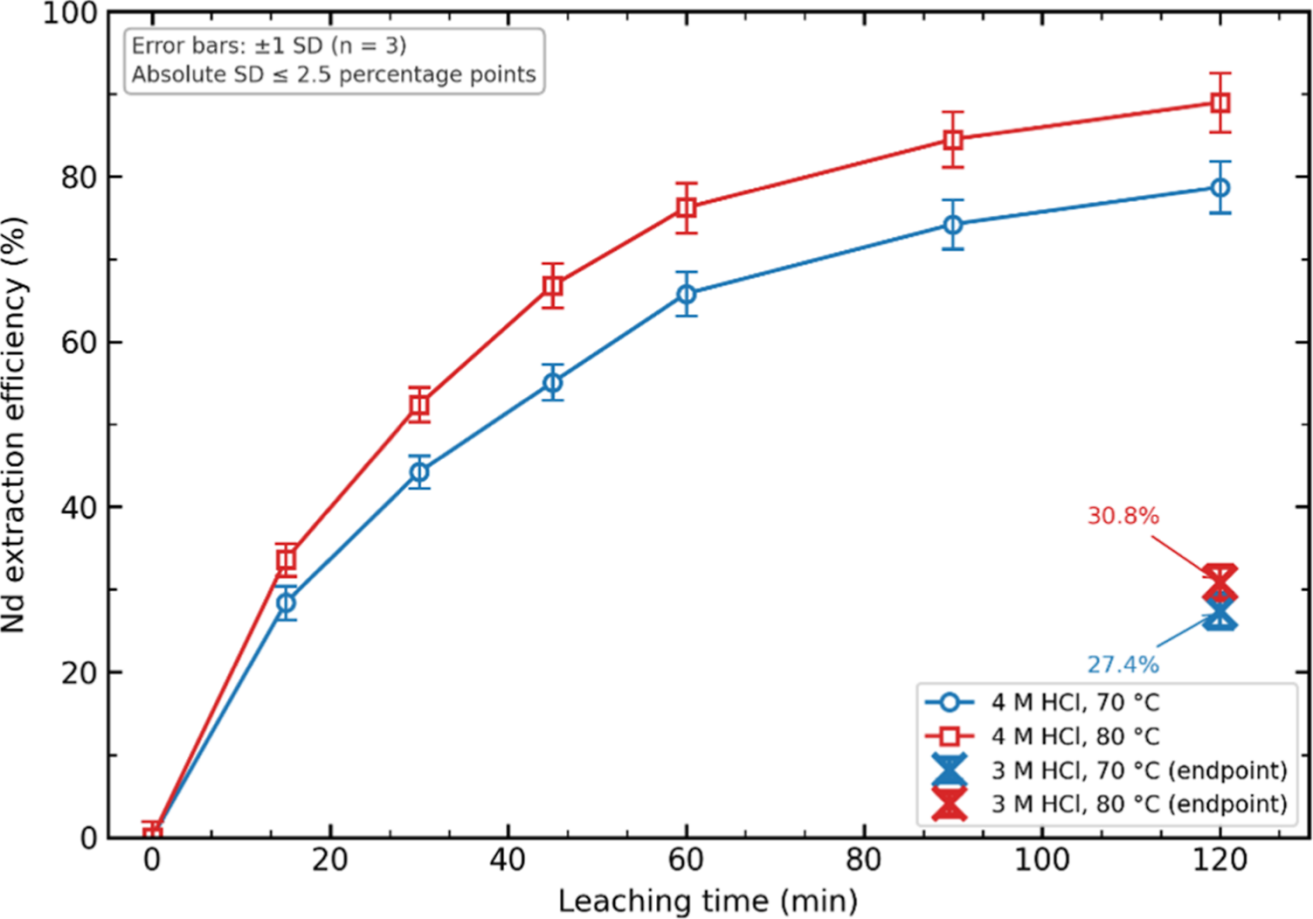
Nd extraction efficiency as a function of leaching time for 3 and 4 M HCl concentrations at 70 and 80 °C (600 °C roasting, +H_2_O_2_). Filled symbols and solid lines represent 4 M HCl (full kinetic curves); cross symbols represent 3 M HCl end point measurements at 120 min. Error bars represent ±1 SD (*n* = 3); absolute SD ≤ 2.5 percentage points. The pronounced difference between 3 and 4 M HCl was confirmed by independent repeat experiments conducted under identical conditions.

**Figure 7:**
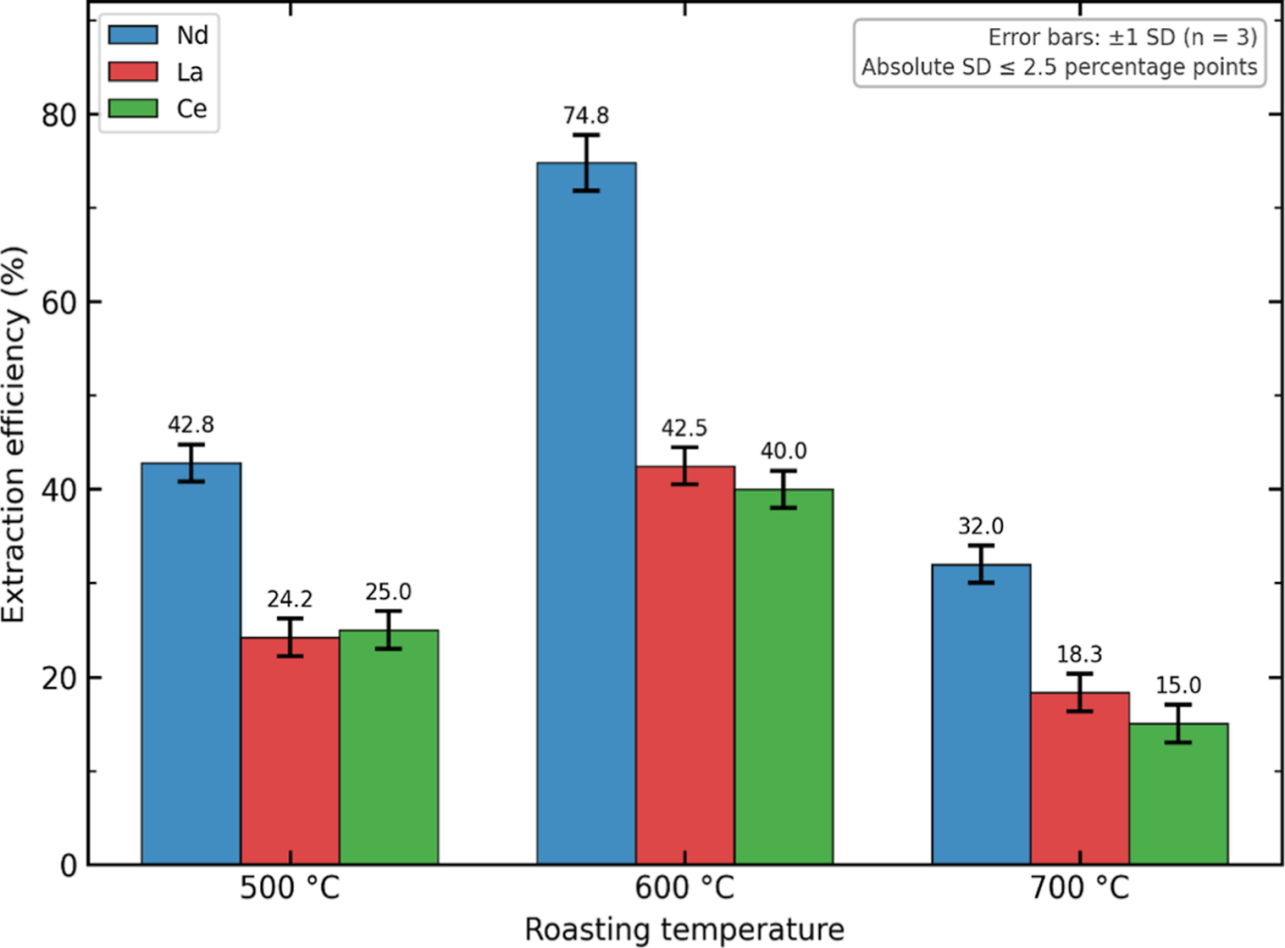
Extraction efficiencies of Nd, La, and Ce as a function of alkali roasting temperature (4 M HCl + H_2_O_2_, 80 °C, 120 min). The marked decline at 700 °C is consistent with partial sintering and surface passivation of the roasted calcine. Error bars represent ±1 SD (*n* = 3); absolute SD ≤ 2.5 percentage points.

**Figure 8:**
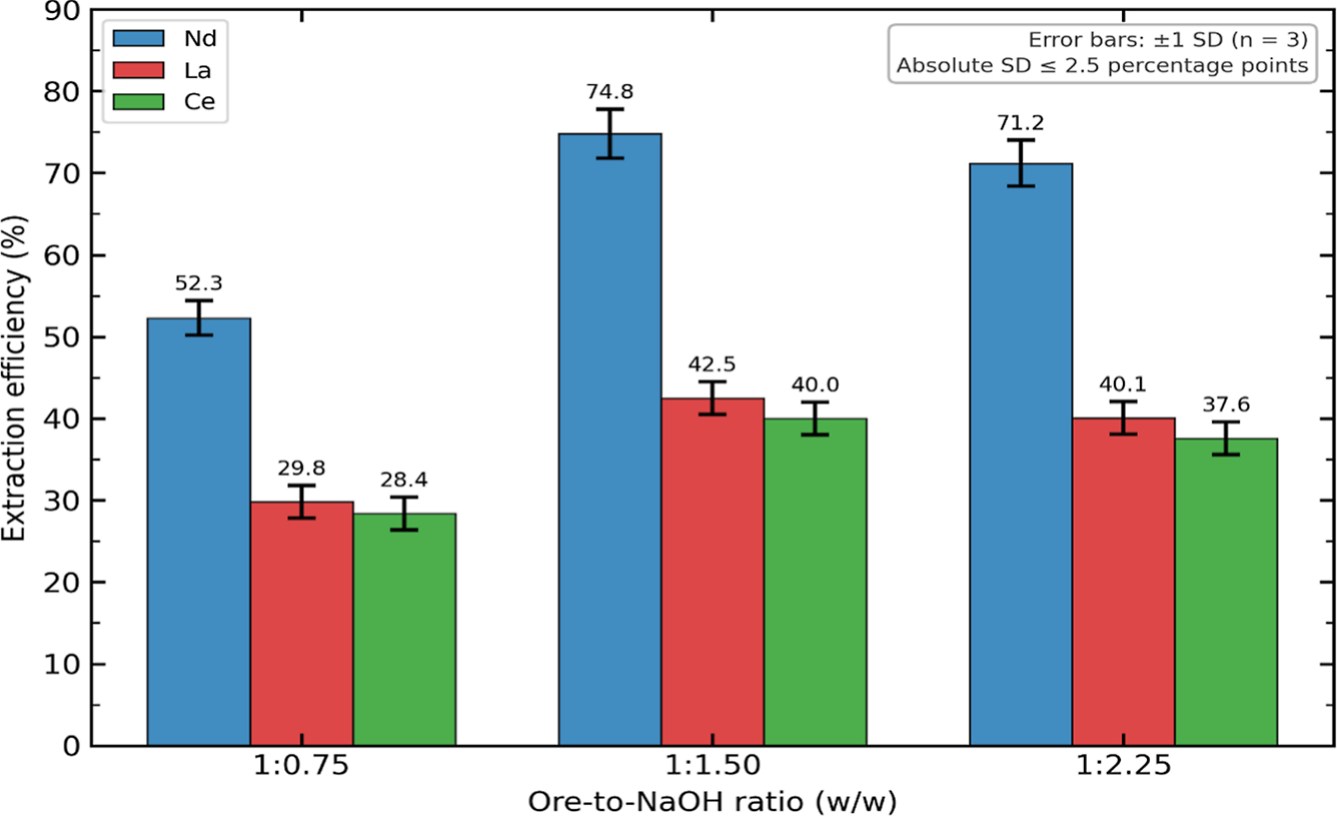
Extraction efficiencies of Nd, La, and Ce as a function of ore-to-NaOH ratio (w/w) at 600 °C roasting (4 M HCl + H_2_O_2_, 80 °C, 120 min). The 1:1.5 ratio was identified as the operational optimum, providing acceptable REE extraction efficiency while avoiding excessive silicate dissolution and gelation. Error bars represent ±1 SD (*n* = 3); absolute SD ≤ 2.5 percentage points.

**Figure 9:**
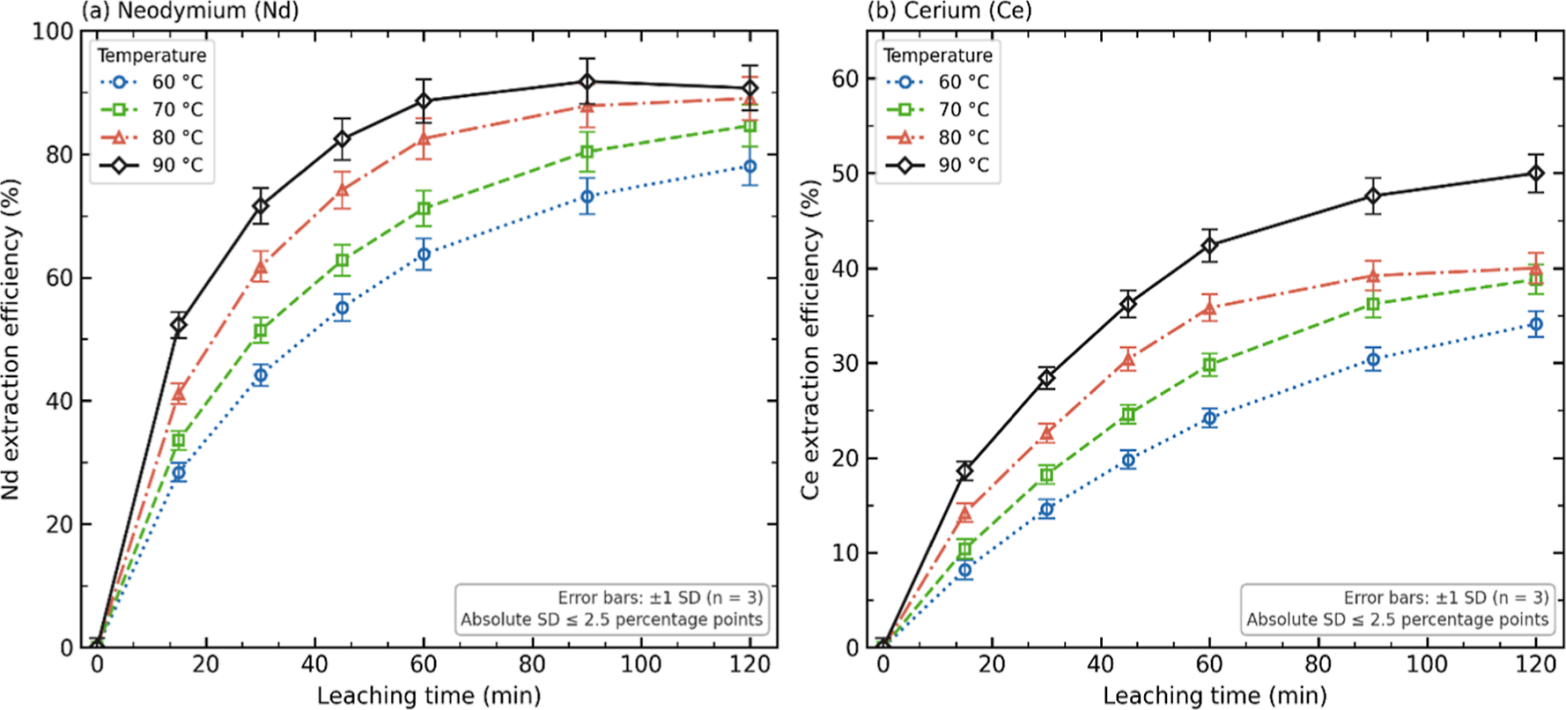
Effect of leaching temperature on the dissolution kinetics of (a) neodymium (Nd) and (b) cerium (Ce) under optimized conditions (4 M HCl + H_2_O_2_, 120 min). The divergent extraction behavior between Nd and Ce reflects the selective separation advantage of the alkali roasting–reductive leaching route. Error bars represent ±1 SD (*n* = 3); absolute SD ≤ 2.5 percentage points.

**Figure 10:**
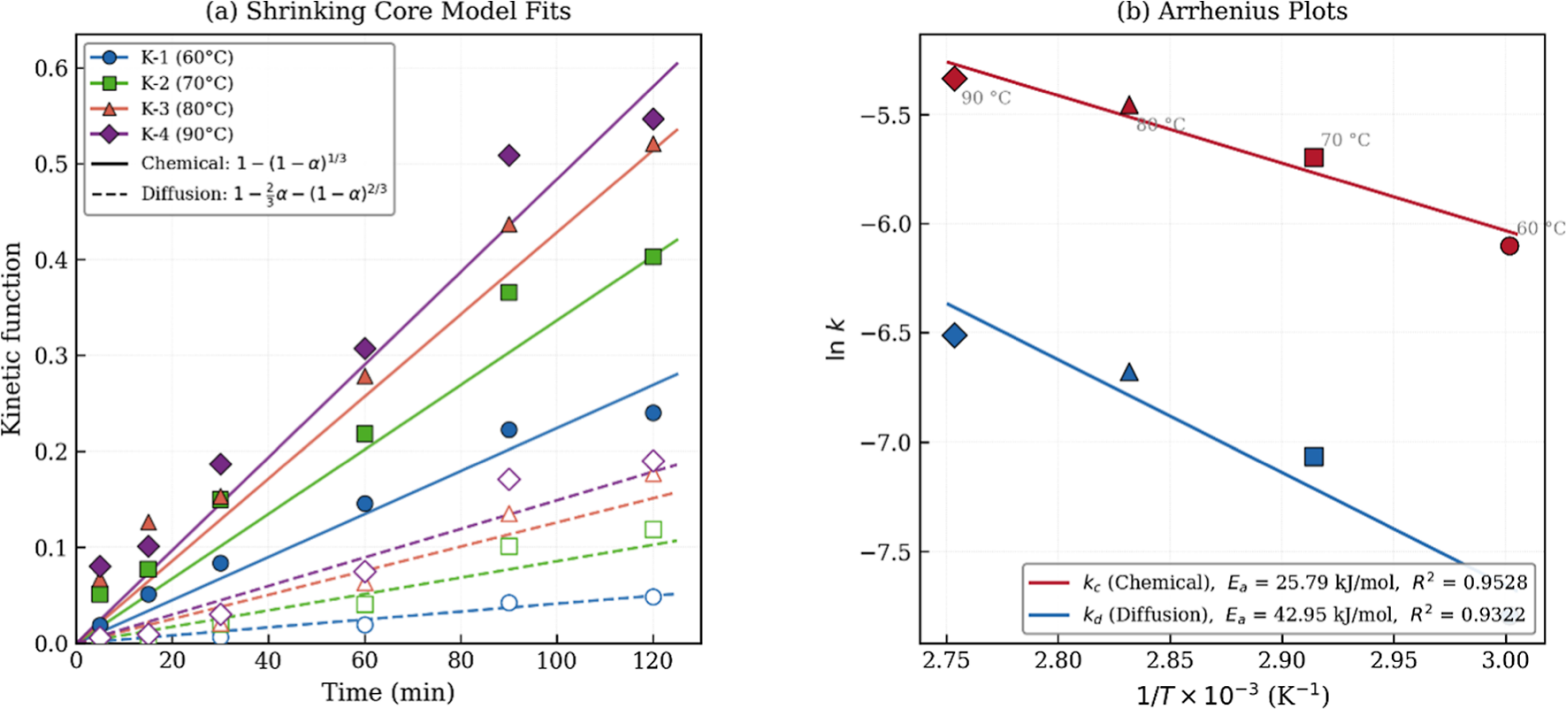
Kinetic analysis of neodymium (Nd) dissolution. (a) Linearized plots of the surface chemical reaction control model (solid lines, filled symbols) and the product layer diffusion control model (dashed lines, open symbols) as a function of time at different temperatures. The comparable fits of both models suggest a mixed-control dissolution mechanism. (b) Arrhenius plots for the surface chemical reaction (*k*_c) and product layer diffusion (*k*_d) rate constants. The calculated activation energies (*E*_a_ = 25.79 and 42.95 kJ/mol, respectively) and the high linearity of both fits further support a surface reaction-dominant mixed-control regime. Error bars in panel (a) represent ±1 SD (*n* = 3).

**Figure 11:**
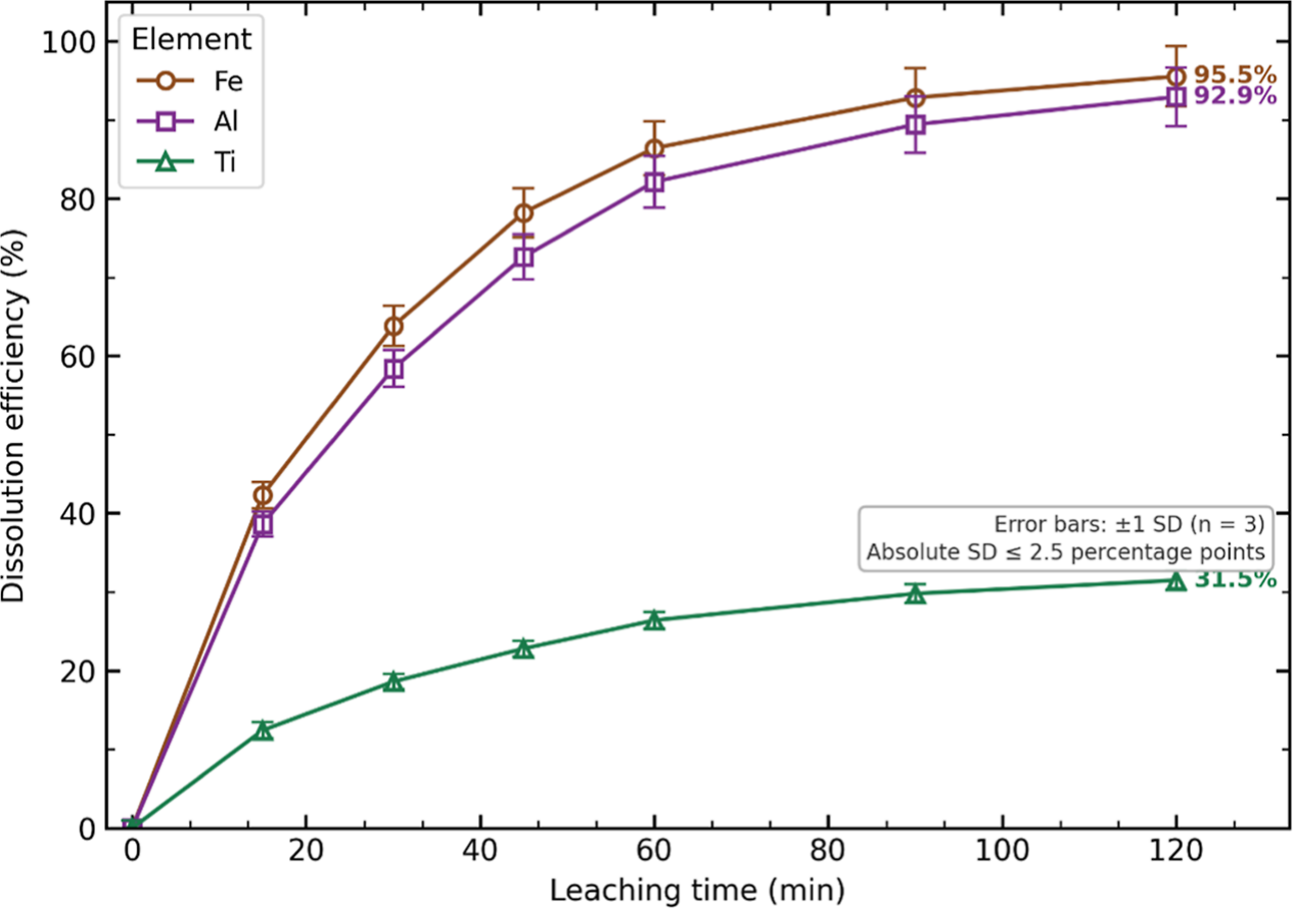
Dissolution behavior of impurity elements (Fe, Al, and Ti) as a function of leaching time under optimized conditions (4 M HCl + H_2_O_2_, 90 °C, 120 min). The limited Ti extraction (∼31.5%) contrasts with the near-complete dissolution of Fe and Al, highlighting the natural selectivity of the process against titanium. Error bars represent ±1 SD (*n* = 3); absolute SD ≤ 2.5 percentage points.

**Figure 12:**
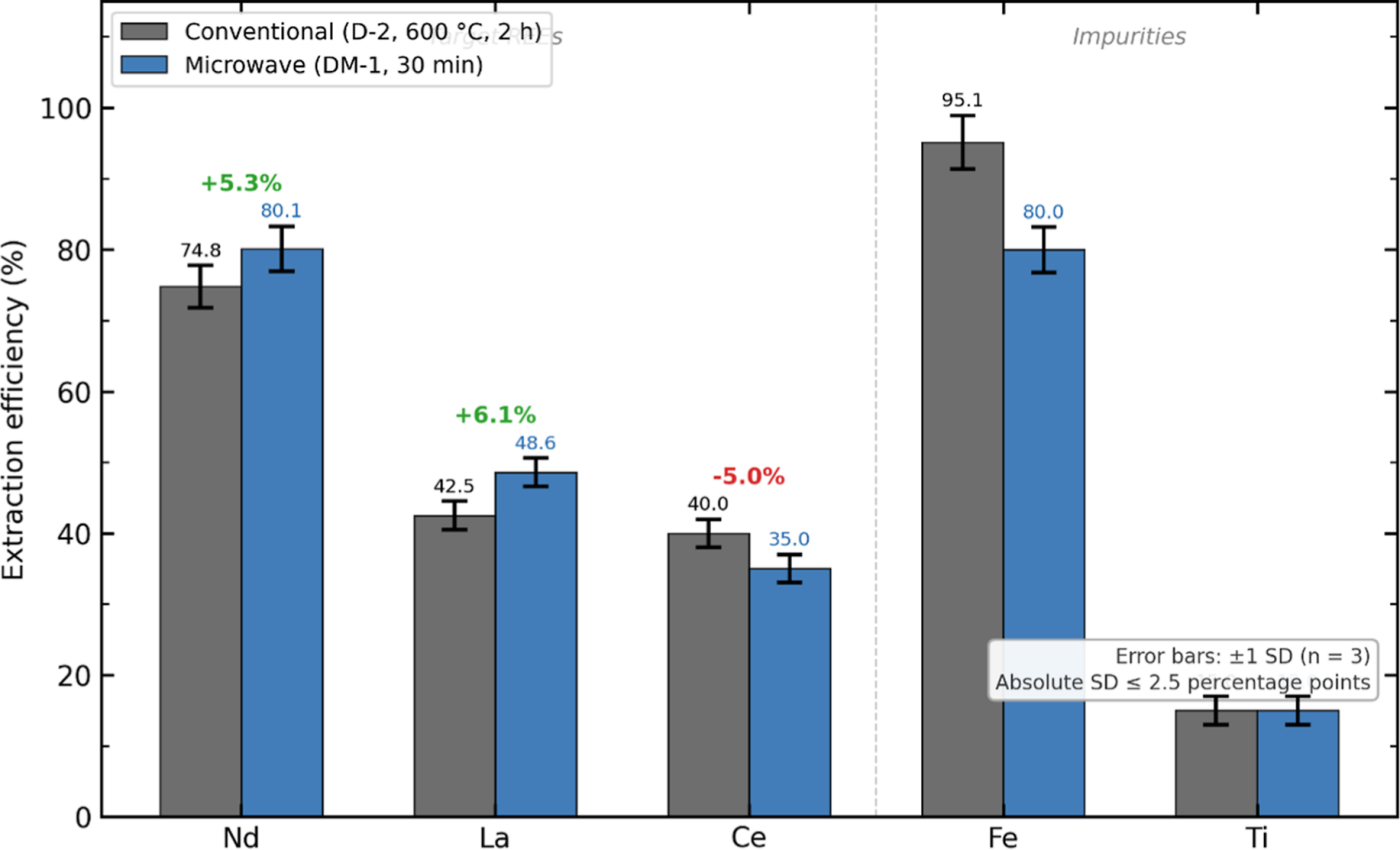
Comparison of extraction efficiencies (%) obtained by conventional furnace roasting (D-2, 600 °C, 2 h) and microwave-assisted roasting (DM-1, 30 min; 15 min ramp +15 min hold), followed by reductive acid leaching (4 M HCl + H_2_O_2_, 120 min, 80 °C). Microwave treatment enhanced Nd and La extraction efficiencies by +5.3 and +6.1 percentage points, respectively, while Ce extraction decreased slightly (−5.0%), suggesting that the microwave-assisted thermal treatment may have promoted further oxidation of Ce-bearing species. Fe and Ti represent codissolved impurity elements. Error bars represent ±1 SD (*n* = 3); absolute SD ≤ 2.5 percentage points.

**Figure 13:**
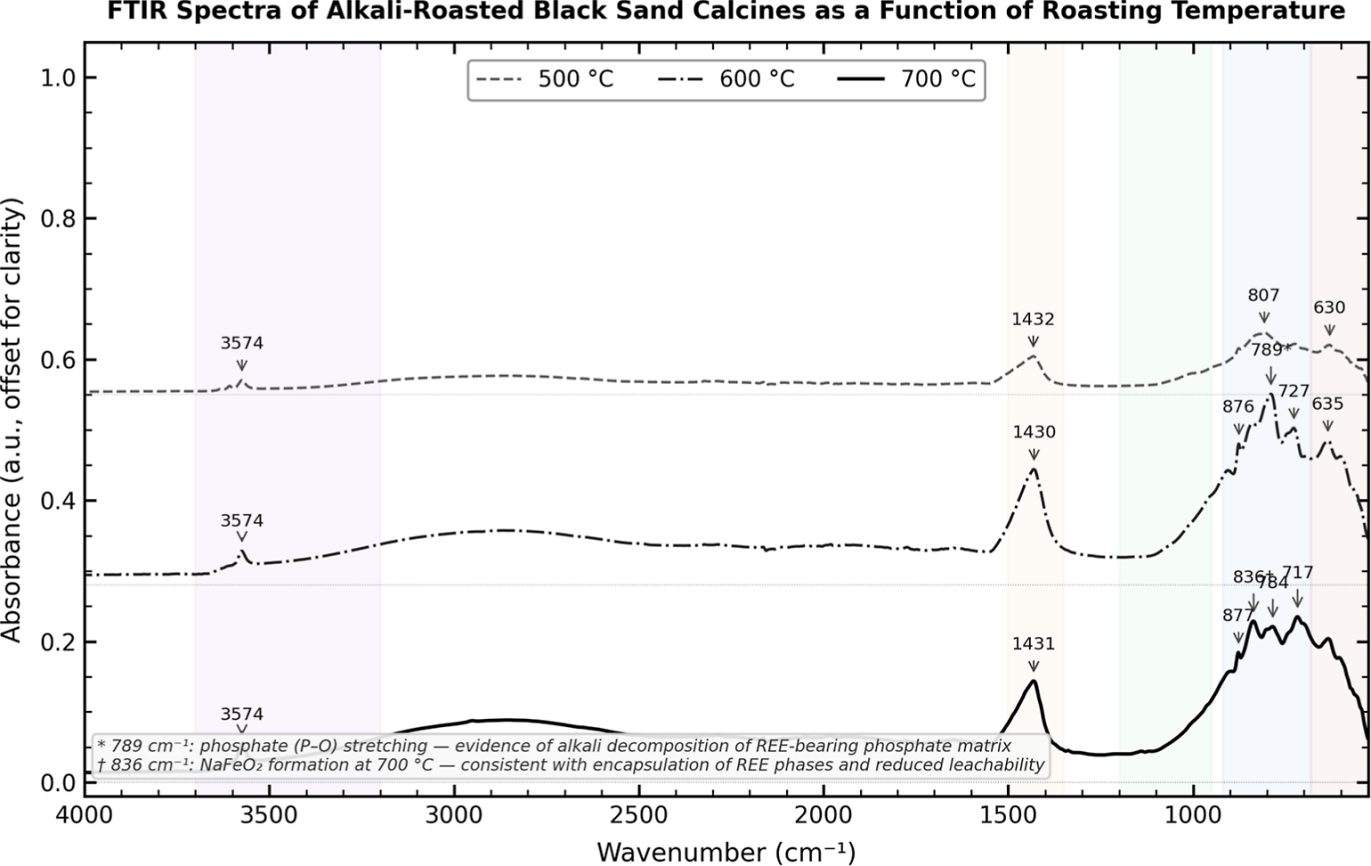
FTIR spectra of alkali-roasted black sand calcines as a function of roasting temperature (500, 600, and 700 °C). Spectra are offset vertically for clarity. Key bands: 630–635 cm^–1^ (Fe–O stretching, hematite); 727–789 cm^–1^ (P–O stretching consistent with sodium phosphate phases, indicative of REE-bearing phosphate matrix structural activation); 836 cm^–1^ (consistent with NaFeO_2_, emerging at 700 °C only); 876–877 cm^–1^ (CO_3_^2–^); 1430–1432 cm^–1^ (CO_3_^2–^ asymmetric stretching); and 3574 cm^–1^ (O–H stretching, consistent with REE hydroxide species). The dramatic increase in absorbance intensity between 500 and 600 °C supports effective phase transformation of the matrix at the optimal roasting temperature, while the emergence of the 836 cm^–1^ band at 700 °C provides spectroscopic support for refractory phase formation and REE encapsulation at excessive temperatures.

**Figure 14:**
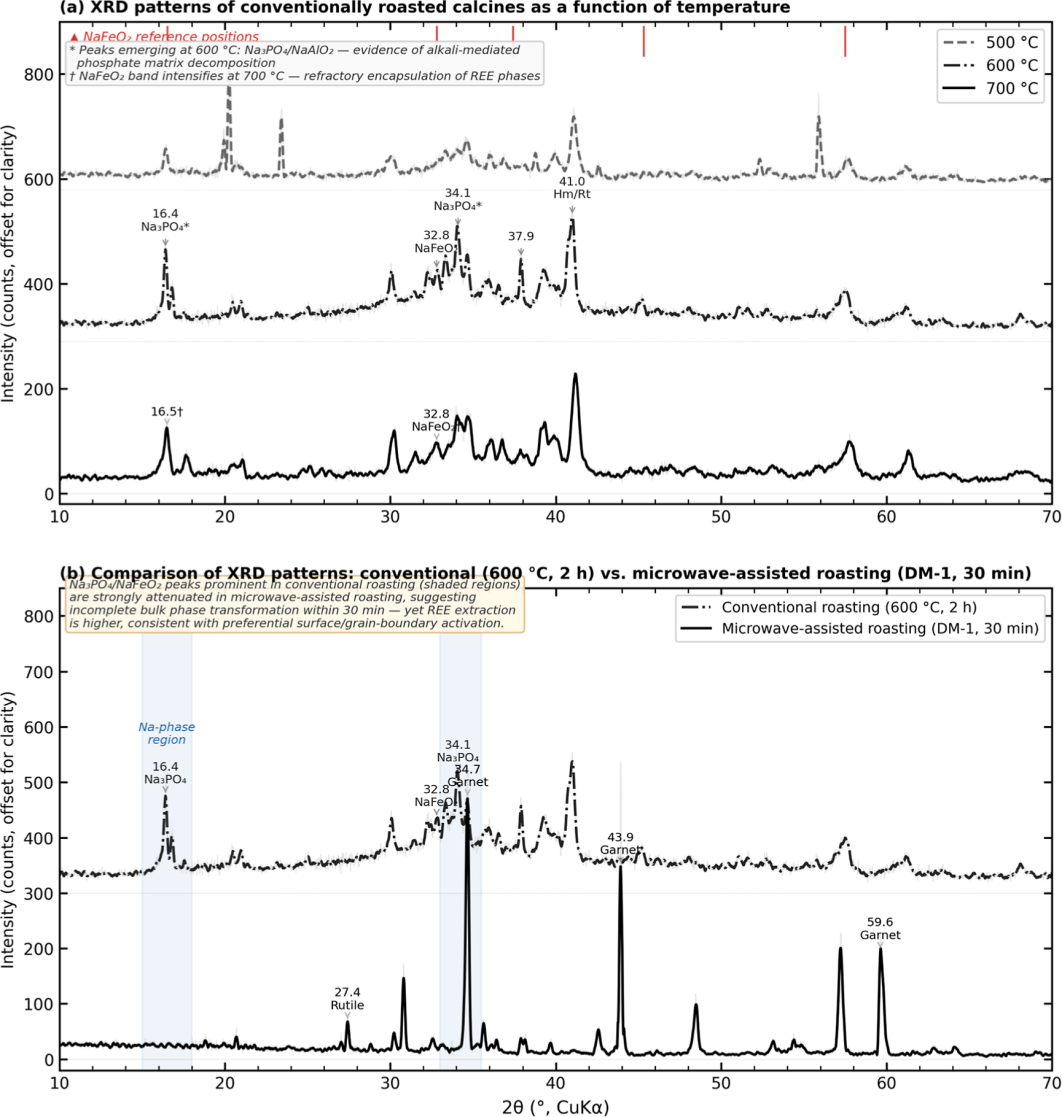
XRD patterns of alkali-roasted calcines. (a) Conventional furnace roasting at 500, 600, and 700 °C (patterns offset vertically for clarity). Key features: absence of new crystalline phases at 500 °C; emergence of peaks at 2θ = 16.4° and 34.1° at 600 °C, consistent with Na_3_PO_4_/NaAlO_2_ formation and alkali-mediated structural activation; and intensification of reflections at 16.5° and 32.8° at 700 °C, consistent with NaFeO_2_ formation and REE encapsulation. (b) Comparison of conventionally roasted (600 °C, 2 h) and microwave-assisted roasted (DM-1, 30 min) calcines. The microwave-roasted product retains garnet-dominated diffraction peaks (34.7°, 43.9°, 59.6°) with weakly developed Na-phase reflections, suggesting less complete bulk phase transformation relative to conventional roasting yet yielding higher REE extraction efficiencies—consistent with preferential surface and grain-boundary activation under microwave irradiation. Shaded regions highlight Na-phase diagnostic positions.

Given the significant thorium content of the feedstock utilized in this study, it is inferred that the monazite phase—if present—may have undergone metamictization to some degree, potentially rendering it partially or predominantly amorphous and thereby attenuating its signature in the XRD diffractogram. Furthermore, because amorphous or structurally disordered phases are generally considered to exhibit heightened susceptibility to chemical dissolution compared to their highly crystalline counterparts, this structural condition may confer a potential kinetic benefit for the subsequent leaching stage, though this remains to be confirmed.^24,45^ The potential kinetic advantage associated with metamictization can be interpreted in terms of structural disorder and excess free energy. Radiation-induced amorphization disrupts the long-range periodicity of the mineral lattice and may generate defects, strained coordination environments, and localized structural instability. Such disorder is generally expected to reduce the resistance of the mineral to chemical attack relative to a highly crystalline counterpart by lowering the thermodynamic stability of the surface and increasing the density of reactive sites available for alkali or acid attack.^45^ While the kinetic consequences of metamictization have been more extensively documented for zircon (ZrSiO_4_),^45^ the structural parallels between zircon and monazite (CePO_4_)—both orthosilicate/orthophosphate minerals with comparable radiation damage susceptibility and similar mechanisms of alpha-decay-induced lattice disruption—suggest that a similar reactivity enhancement may apply to the REE-bearing phosphate phase in the present sample. In this context, the inferred partially metamict nature of the REE-bearing phosphate phase in the Sart sample may have contributed to its susceptibility to structural activation during roasting at 600 °C. Nevertheless, this interpretation remains a working hypothesis in the absence of direct microstructural confirmation and should be regarded as a plausible mechanistic suggestion rather than an established conclusion.

### 2.2 Optical and Ore Microscopy (Petrographic Investigation)

To conduct a comprehensive examination of the textural properties, degree of liberation, and morphological characteristics of the mineralogical phases identified via XRD, representative thin and polished sections of the sample were prepared. Examinations under a transmitted-light polarizing microscope revealed that the transparent heavy mineral assemblage is predominantly composed of garnet, rutile, and zircon, accompanied by minor amounts of feldspar, quartz, and epidote, alongside trace quantities of magnesite, pyroxene ± amphibole, titanite, kyanite, and olivine.^12,40^ Observations under cross-polarized light (XPL) demonstrated the characteristic isotropic nature of the garnets (appearing dark), whereas minerals such as zircon and epidote were distinctly identified by their high relief and high-order interference colors. Under plane-polarized light (PPL), garnets and opaque minerals exhibited sharply defined grain boundaries, and rutile grains were readily distinguishable within the matrix due to their distinctive brown coloration (Figure 2a,b).

Complementary investigations of the polished sections utilizing reflected-light ore microscopy elucidated the structural features of the opaque ore minerals. Hematite (Fe_2_O_3_) was identified as the most abundant opaque phase, followed successively by goethite and ilmenite (FeTiO_3_) (Figure 2c,d). Textural analyses suggest that a significant proportion of the hematite occurs as pseudomorphs, indicative of martitization of primary magnetite grains (i.e., hematite pseudomorphous after magnetite).^14,23^ Furthermore, residual microcores of unoxidized magnetite (FeO·Fe_2_O_3_) were occasionally detected within certain hematite grains. Among the other opaque constituents, goethite exhibited subhedral to concentric-textured morphologies, while ilmenite was observed as slightly elongated, tabular grains.^11^ Rutile, exhibiting partial reflectance, was distinguished as light-gray grains displaying strong internal reflections. Conversely, silicate-based transparent minerals, which are nonreflective due to their inherent optical properties, were collectively classified as “other minerals” (OM). In addition, trace particles of manganese oxide and chromium oxide were detected within the bulk matrix.

### 2.3 Experimental Procedure

#### 2.3.1 Sample Preparation and Alkali Roasting

To decompose the refractory structure of the monazite mineral and liberate the rare earth elements (REEs), an alkali roasting (NaOH decomposition) method was adopted. This approach is favored over conventional acid baking as it represents a more environmentally benign alternative that additionally facilitates the recovery of phosphate as a valuable byproduct. Prior to the experiments, the heavy mineral concentrate was milled to a particle size of <53 μm to maximize the solid-phase reaction surface area.

All leaching experiments were performed in a 500 mL three-necked glass reactor equipped with a magnetic stirrer and a reflux condenser to prevent evaporative loss of the lixiviant. To maintain a constant hydrodynamic environment, the stirring speed was fixed at 300 rpm throughout all trials, ensuring adequate suspension of particles while minimizing mechanical attrition. The solid-to-liquid (S/L) ratio was maintained at a constant 1:10 (*w*/*v*) (e.g., 20 g of roasted calcine per 200 mL of acid) to ensure sufficient proton availability and to prevent saturation effects during the dissolution process.

Technical grade sodium hydroxide (NaOH) was utilized as the reagent for the roasting process. To systematically evaluate the effects of the process parameters and heating mechanisms on reaction efficiency, two distinct roasting protocols were implemented:

##### 2.3.1.1 Conventional Furnace Roasting

To maximize the homogeneity of the solid–solid mixture, a “wet impregnation” technique, as recommended by Pereira et al.,^8^ was employed. Predetermined ratios of NaOH were dissolved in a minimal volume of deionized water, impregnated into the sample, dried at 105 °C, and subsequently transferred to nickel crucibles for roasting. The roasting temperature and duration were maintained at 600 °C and 2 h, respectively, which were identified as the conditions providing the most effective structural activation of the REE-bearing matrix for subsequent acid leaching. To investigate the effect of the stoichiometric ratio, three different ore-to-NaOH mass ratios (w/w) were tested: 1:0.75 (Set B-1), 1:1.50 (Set ref), and 1:2.25 (Set B-3). This range was established based on the stoichiometric NaOH requirement for complete decomposition of a monazite-type phosphate matrix (3NaOH per PO_4_ unit, corresponding to approximately 1:1 w/w for the bulk ore), with the lower bound (1:0.75) representing a substoichiometric condition and the upper bound (1:2.25) representing a 50% excess above the midpoint, consistent with the ranges reported in the monazite alkali roasting literature.^8,32,44^

##### 2.3.1.2 Microwave-Assisted Roasting

In line with the process intensification approach, the effect of microwave irradiation on roasting efficiency was investigated (Set DM). Microwave-assisted roasting was performed in a temperature-controlled microwave system using a programed heating schedule with a total treatment time of 30 min, consisting of a 15 min ramp stage followed by a 15 min holding stage. The sample–NaOH mixture was treated under automatic power modulation, whereby the applied microwave power was continuously adjusted by the instrument to maintain the preset roasting conditions, rather than being applied as a fixed nominal power input. After microwave treatment, the roasted cake was subjected to water washing (100 mL deionized water at 60 °C) and filtration to remove water-soluble species and excess alkali. The resulting solid residue was then leached in the selected acid medium with or without H_2_O_2_ under the conditions specified in Table 4.

**Table 4:** Summary of the Experimental Design and Operational Parameters Employed for the Parametric Optimization and Kinetic Analysis of the Hydrometallurgical Process

Exp. Code	Investigated Parameter	Stage 1: Roasting Conditions	Stage 2: Water Leaching	Stage 3: Acid Leaching Conditions	Leaching Temp./Time
D-1	Effect of Roasting Temp.	5 g Sample +7.5 g NaOH (500 °C)	100 mL Water, 60 °C, Filtration	4 M HCl + H_2_O_2_	80 °C/120 min
D-2	Reference (Baseline)	5 g Sample +7.5 g NaOH (600 °C)	100 mL Water, 60 °C, Filtration	4 M HCl + H_2_O_2_	80 °C/120 min
D-3	Effect of Roasting Temp.	5 g Sample +7.5 g NaOH (700 °C)	100 mL Water, 60 °C, Filtration	4 M HCl + H_2_O_2_	80 °C/120 min
B-1	Effect of NaOH Dosage	5 g Sample +3.75 g NaOH (600 °C)	100 mL Water, 60 °C, Filtration	4 M HCl + H_2_O_2_	80 °C/120 min
B-3	Effect of NaOH Dosage	5 g Sample +11.25 g NaOH (600 °C)	100 mL Water, 60 °C, Filtration	4 M HCl + H_2_O_2_	80 °C/120 min
D-4	Effect of Acid Conc.	600 °C (Same calcine as D-2)	100 mL Water, 60 °C, Filtration	2 M HCl + H_2_O_2_	80 °C/120 min
D-5	Effect of Acid Conc.	600 °C (Same calcine as D-2)	100 mL Water, 60 °C, Filtration	6 M HCl + H_2_O_2_	80 °C/120 min
D-6	Effect of Reductant (H_2_O_2_)	600 °C (Same calcine as D-2)	100 mL Water, 60 °C, Filtration	4 M HCl (No H_2_O_2_)	80 °C/120 min
D-7	Effect of Acid Type	600 °C (Same calcine as D-2)	100 mL Water, 60 °C, Filtration	4 M HNO_3_ + H_2_O_2_	80 °C/120 min
D-8	Effect of Acid Type	600 °C (Same calcine as D-2)	100 mL Water, 60 °C, Filtration	4 M H_2_SO_4_ + H_2_O_2_	80 °C/120 min
DM-1	Microwave Effect	5 g Sample +7.5 g NaOH (Microwave, 30 min: 15 min ramp +15 min hold)	100 mL Water, 60 °C, Filtration	4 M HCl + H_2_O_2_	80 °C/120 min
DM-2	Microwave Effect	5 g Sample +7.5 g NaOH (Microwave, 30 min: 15 min ramp +15 min hold)	100 mL Water, 60 °C, Filtration	4 M HCl (No H_2_O_2_)	80 °C/120 min
K-1	Leaching Kinetics (Temp)	600 °C (Same calcine as D-2)	100 mL Water, 60 °C, Filtration	4 M HCl + H_2_O_2_	60 °C/5–120 min
K-2	Leaching Kinetics (Temp)	600 °C (Same calcine as D-2)	100 mL Water, 60 °C, Filtration	4 M HCl + H_2_O_2_	70 °C/5–120 min
K-3	Leaching Kinetics (Temp)	600 °C (Same calcine as D-2)	100 mL Water, 60 °C, Filtration	4 M HCl + H_2_O_2_	80 °C/5–120 min
K-4	Leaching Kinetics (Temp)	600 °C (Same calcine as D-2)	100 mL Water, 60 °C, Filtration	4 M HCl + H_2_O_2_	90 °C/5–120 min
K-2 (3 M)	Acid Conc. and Temp. interaction	600 °C (Same calcine as D-2)	100 mL Water, 60 °C, Filtration	3 M HCl + H_2_O_2_	70 °C/120 min
K-3 (3 M)	Acid Conc. and Temp. Interaction	600 °C (Same calcine as D-2)	100 mL Water, 60 °C, Filtration	3 M HCl + H_2_O_2_	80 °C/120 min

#### 2.3.2 Water Leaching and Impurity Removal

Following the roasting process, the resultant cake (calcine) was subjected to water leaching to eliminate water-soluble sodium phosphate (Na_3_PO_4_) and unreacted excess alkali. To preclude the peptization of rare earth hydroxides and ensure optimal phosphate removal, the operational parameters were optimized in accordance with the methodologies suggested by Wang et al.^41^ As a standard protocol, the process was conducted by dispersing 5 g of the roasted cake in 100 mL of deionized water, followed by continuous mechanical stirring at 60 °C for 30 min. The isolation of this purified solid residue constitutes a critical beneficiation step that significantly upgrades the REE concentration prior to the subsequent acid leaching phase, a strategic advantage corroborated by Sosidi et al.^32^

#### 2.3.3 Reductive Acid Leaching and Kinetic Studies

The dissolution of REEs from the water-leached cake was conducted in a thermostatic glass reactor equipped with a reflux condenser and a mechanical stirrer operating at a constant agitation speed of 300 rpm. Hydrochloric acid (HCl) was selected as the lixiviant in preference to sulfuric acid owing to its superior potential to enhance REE solubility through the formation of stable aqueous chloride complexes, thereby circumventing the precipitation of insoluble double-sulfate salts.^31^ During the experimental trials, key operational parameters, specifically acid concentration (3 and 4 M) and temperature (60–90 °C), were systematically optimized.

The most critical phase of the dissolution process involves the recovery of cerium, which inadvertently oxidizes into the highly acid refractory cerium dioxide (CeO_2_) phase during the preceding alkali roasting stage. To overcome this metallurgical challenge, the reductive leaching mechanism discussed by Zhang et al.^44^ was adopted as the foundational strategy. Accordingly, two distinct leaching conditions were investigated: (i) control group (DM-2): standard acid leaching in the absence of any reducing additives; (ii) reductive leaching (DM-1 and K-series): hydrogen peroxide (H_2_O_2_, 30% v/v) was introduced into the lixiviant as a reducing agent to convert insoluble Ce^4+^ species into the highly soluble Ce^3+^ form, thereby improving overall leaching recovery.^10,44^

To delineate the reaction kinetics and mathematically identify the rate-controlling step of the dissolution process, the Shrinking Core Model (SCM)—successfully implemented by Sun et al.^35^ for the kinetic analysis of metallurgical wastes—was employed. Representative aliquots of the leach liquor were periodically withdrawn at 5, 15, 30, 60, 90, and 120 min intervals for quantitative elemental analysis.

#### 2.3.4 Data Calculation and Kinetic Modeling

The extraction efficiency of the rare earth elements (ηREE), which serves as the primary quantitative indicator of the leaching performance, was calculated using eq 1. This efficiency is defined as the mass ratio of the solubilized metal in the pregnant leach solution (PLS) to the initial metal content present in the solid feedstock

1 η=(C×V)/(M×ω)×100

where η represents the extraction efficiency of the targeted rare earth element (%), *C* is the metal concentration in the filtrate (mg/L), *V* is the volume of the lixiviant (L), *M* is the mass of the solid sample (mg), and ω is the mass fraction of the metal in the initial sample.

To determine the reaction kinetics and identify the rate-controlling step, the Shrinking Core Model (SCM), widely implemented for heterogeneous solid–liquid reactions, was employed.^35^ Assuming the solid particles are predominantly spherical, the SCM postulates that the overall dissolution rate is predominantly controlled by either the surface chemical reaction or mass transfer (diffusion) through a porous product layer.^46^

$$1-{\left(1-X\right)}^{1/3}=k_{C}\cdot t$$ 2

$$1-\left(2/3\right)X-{\left(1-X\right)}^{2/3}=k_{D}\cdot t$$ 3

where *X* represents the fractional conversion of the targeted REEs (η/100), *t* is the reaction time (min), *k*_C_ is the apparent rate constant for the surface chemical reaction control model (min^–1^), and *k*_D_ is the apparent rate constant for the product layer diffusion control model (min^–1^). The apparent activation energy (*E*_a_) was determined using the Arrhenius equation

4 $$\ln k=\ln A-E_{a}/\left(R\cdot T\right)$$

where *A* denotes the pre-exponential (frequency) factor, *R* represents the universal gas constant (8.314 J/mol·K), and *T* indicates the absolute operational temperature (K).

### 2.4 Analytical Techniques

All chemical reagents used in this study, including hydrochloric acid (HCl, 37%), nitric acid (HNO_3_, 65%), and hydrogen peroxide (H_2_O_2_, 30%), were of analytical grade (Sigma-Aldrich or Merck) and were used without further purification. The elemental concentrations in the pregnant leach solutions (PLS) were determined using inductively coupled plasma optical emission spectroscopy (ICP-OES, Agilent 5110) and ICP–MS (Agilent 7900) for trace rare earth elements.^43^ The analytical instruments were calibrated using multielement standard solutions (Inorganic Ventures) with correlation coefficients (*R* ^2^) exceeding 0.999. The limit of detection (LOD) for REEs was determined to be in the range of 0.01–0.1 μg/L.

To ensure the reliability and reproducibility of the data, all experiments and instrumental analyses were performed in triplicate (*n* = 3), and mean values are reported throughout. The precision of all reported extraction efficiencies was assessed by calculating the relative standard deviation (RSD), which remained below 5% in all cases. The corresponding absolute standard deviation (SD) values are typically ≤2.5 percentage points across all experimental sets. Error bars in all figures represent ±1 SD (*n* = 3) of the triplicate measurements; where error bars are not visually discernible, the SD is smaller than the symbol size. The microwave experiments were conducted in a temperature-controlled microwave system operating under automatic power modulation with a programed heating schedule (30 min total: 15 min ramp +15 min hold).

## 3 Results and Discussion

### 3.1 Roasting Product and Impurity Removal

Following the alkali roasting process, during the hot water leaching of the resultant calcine, a distinct color transition of the filtrate to an emerald-green hue was observed (Figure 3a). Consistent with the established hydrometallurgical literature, this characteristic coloration is directly attributed to the oxidation of manganese within the ore matrix during roasting, resulting in the formation of highly water-soluble sodium manganate (Na_2_MnO_4_).^24,32^ This observation is consistent with the selective removal of major impurities, such as manganese, from the system, thereby serving as an effective preconcentration step prior to the targeted acid leaching of rare earth elements.

This stage is considered to be critical to the overall efficacy of the hydrometallurgical process. The effective decantation and elimination of the green filtrate ensure the simultaneous removal of three critical components from the system: the phosphate (PO_4_^3–^) ions liberated from the decomposed monazite lattice, the manganese (Mn) impurities that could otherwise compromise the purity of the final product, and the unreacted excess NaOH. Consequently, the residual brown filter cake, as depicted in Figure 3b, is significantly enriched in both REE and iron hydroxides, rendering it well-prepared as the feedstock for the subsequent acid leaching stage.

### 3.2 Optimization of Leaching Parameters

#### 3.2.1 Effect of Acid Type on Leaching Efficiency

To determine the optimal dissolution conditions for the purified calcine obtained following the alkali roasting stage, comparative acid leaching experiments (Sets D-2, D-7, and D-8) were conducted using hydrochloric acid (HCl), nitric acid (HNO_3_), and sulfuric acid (H_2_SO_4_) at isomolar concentrations (4 M) under standardized operational conditions (80 °C, 120 min). The quantitative effects of the distinct structural properties and reactivities of these acidic lixiviants on the extraction efficiencies of the target light rare earth elements (LREEs: Nd, La, and Ce) are illustrated in Figure 4.^48^

An analysis of the extraction data reveals that the highest elemental dissolution efficiencies were achieved in the HCl medium (set D-2), yielding 74.9% for Nd and 42.5% for La. Under identical operational parameters, the application of HNO_3_ (set D-7) maintained a comparable Nd recovery of 64.1%; however, the extraction efficiency of cerium (Ce) declined to 10.0%. This decline is primarily attributed to the strongly oxidizing nature of nitric acid, coupled with the fact that nitrate ions form thermodynamically weaker aqueous complexes with rare-earth cations than their chloride counterparts.^48^

The most pronounced deterioration in extraction efficiency was observed in the H_2_SO_4_ medium (Set D-8), where the Nd and La recoveries fell to 16.3% and 12.2%, respectively. From a hydrometallurgical perspective, this yield loss is governed by the “double-sulfate precipitation” phenomenon.^31^ The high concentration of sodium (Na^+^) ions introduced during the preceding alkali roasting stage, combined with endogenous calcium (Ca^2+^) ions from the ore matrix, react with the solubilized rare earth elements in the sulfate environment, precipitating highly insoluble sodium-rare earth double sulfate salts (e.g., NaRE­(SO_4_)_2_). Furthermore, the concurrent precipitation of a calcium-based gypsum (CaSO_4_·2H_2_O) sludge rendered solid–liquid separation (filtration) physically challenging and also encapsulated the remaining REEs via coprecipitation mechanisms.^24,31^

5 $${RE}^{3+}+{Na}^{+}+{{2\text{SO}}_{4}}^{2-}\to NaRE{\left({\text{SO}}_{4}\right)}_{2}$$

In light of these empirical findings, hydrochloric acid (HCl) was identified as the most favorable lixiviant under the tested conditions for the subsequent stages of the hydrometallurgical process.

#### 3.2.2 The Role of Hydrogen Peroxide (H_2_O_2_) and Redox Potential

During the reductive acid leaching process subsequent to the alkali roasting stage, the influence of hydrogen peroxide (H_2_O_2_) addition on both the redox potential of the system and the elemental dissolution efficiencies was systematically investigated via comparative experiments (Sets D-2 and D-6). Both experimental trials were executed under isothermal conditions (80 °C) for 120 min, utilizing 4 M HCl as the primary lixiviant. The quantitative impact of incorporating H_2_O_2_ as a reducing agent into the leaching medium on the extraction efficiencies of the target light rare earth elements (LREEs: Nd, La, and Ce) is illustrated in Figure 5.^10,44^

An examination of the experimental findings reveals that the introduction of H_2_O_2_ into the leaching medium exerted no discernible impact on the dissolution efficiencies of neodymium (Nd), iron (Fe), and aluminum (Al); the respective extraction efficiencies remained consistent across both trials. In stark contrast, the addition of H_2_O_2_ produced a markedly different effect on cerium (Ce). In terms of elemental concentration in the pregnant leach solution (PLS), the Ce concentration increased from approximately 130 mg/kg in the absence of H_2_O_2_ to approximately 520 mg/kg upon its addition—a roughly 4-fold increase. Expressed as extraction efficiency, this corresponds to an increase from approximately 10% (Set D-6, no H_2_O_2_) to approximately 40% (Set D-2, with H_2_O_2_) under the same leaching conditions. This highly specific behavior is attributed to the inherent redox sensitivity of cerium.

Consistent with corroborating studies in the hydrometallurgical literature, during the oxidative alkali roasting phase, cerium is oxidized from its soluble trivalent state (Ce^3+^) to the tetravalent cerianite (CeO_2_) phase, which forms a highly stable lattice structure that is exceedingly refractory to standard acid attack.^44^ The suppressed cerium extraction efficiency observed in the plain HCl medium (Set D-6) is therefore attributed to the pronounced resistance of this thermodynamically stable, refractory CeO_2_ phase against simple acidic dissolution.^49^

6 $${2CeO}_{2}+H_{2}O_{2}+{6H}^{+}\to {2Ce}^{3+}+O_{2}+{4H}_{2}O$$

Mechanistically, the incorporated H_2_O_2_ acts to disrupt the refractory Ce^4+^ crystal lattice, reductively converting the cerium into its highly acid soluble Ce^3+^ state. Nevertheless, the observation that the maximum cerium extraction efficiency plateaus at approximately 40% even in the presence of the reducing agent suggests that the partial reductive dissolution of the cerianite structure facilitates the overall breakdown of the encompassing lanthanide matrix without compromising the inherent selectivity of the process toward neodymium (Nd) extraction.

#### 3.2.3 Effect of Acid Concentration on Leaching Efficiency

##### 3.2.3.1 Stage 1—Screening of the Acid Concentration Range

An initial screening study was conducted at a fixed temperature of 80 °C to identify the operational concentration range for hydrochloric acid leaching. Three acid concentrations were evaluated: 2, 4, and 6 M HCl (Sets D-4, D-2, and D-5, respectively), each under otherwise identical conditions (600 °C roasting, H_2_O_2_ addition, 120 min leaching time). This concentration range was selected to bracket the operationally practical window: 2 M represents a mild condition commonly used for REE hydroxide dissolution, 6 M represents the upper practical limit before excessive corrosion and vapor generation become prohibitive, and 4 M was selected as the midpoint informed by the literature on chloride leaching of alkali-roasted rare earth concentrates.^10,44^

The results of this screening stage revealed a clear concentration threshold effect. At 2 M HCl, proton (H^+^) activity was insufficient to effectively decompose the rare-earth hydroxide and iron oxide phases formed during alkali roasting, yielding significantly low Nd extraction. Conversely, increasing the concentration to 6 M HCl did not produce a statistically meaningful improvement in REE extraction efficiency relative to 4 M HCl while simultaneously generating excessive corrosive vapor and increasing reagent consumption beyond practical limits.

On the basis of these screening results, 4 M HCl was identified as the optimal acid concentration, offering the most favorable balance between the extraction efficiency and operational feasibility. This concentration was therefore adopted as the fixed lixiviant for all of the subsequent experimental sets.

##### 3.2.3.2 Stage 2—Kinetic Threshold Comparison: 3 M vs 4 M HCl

Following the identification of 4 M HCl as the process optimum, a secondary comparative study was performed to mechanistically substantiate the kinetic superiority of this concentration and to quantify the thermodynamic driving force it provides relative to a suboptimal threshold concentration. For this purpose, 3 M HCl was selected as the comparative baseline—a concentration that lies within the technically feasible range yet falls below the identified optimum—and leaching experiments were conducted at two temperatures (70 and 80 °C). The time-dependent variation of Nd extraction efficiency as a function of acid concentration and temperature is illustrated in Figure 6.

The comparative data reveal a pronounced concentration-dependent divergence in leaching kinetics. At 70 °C, 3 M HCl yielded only 27.4% Nd extraction efficiency after 120 min, whereas 4 M HCl under the same thermal conditions achieved 78.7%—a difference of more than 51 percentage points. A consistent trend was observed at 80 °C: 3 M HCl produced a maximum extraction efficiency of 30.8%, while 4 M HCl delivered 89.0% under otherwise identical conditions. The pronounced inefficiency of 3 M HCl is primarily attributed to the insufficient proton (H^+^) activity required to effectively decompose the phases formed during alkali roasting—specifically rare earth hydroxides and iron oxides. The data collectively indicate that a threshold proton activity, achieved at 4 M HCl, is required to overcome the structural resistance of the calcine matrix. Below this threshold, even moderate increases in temperature (from 70 to 80 °C) fail to compensate for the thermodynamic deficit imposed by low acid molarity. These findings align with recent studies on the chloride leaching of refractory rare earth concentrates, which emphasize precise molarity control as a prerequisite for maximizing extraction kinetics.^49^ Because the difference in Nd extraction between 3 and 4 M HCl appeared unusually pronounced, the corresponding leaching experiments were independently repeated under identical roasting, leaching, and analytical conditions. The repeated tests reproduced the same concentration-dependent trend within experimental uncertainty, confirming that the sharp increase in extraction at 4 M HCl was not an isolated analytical or procedural artifact. Instead, the results support the interpretation of a threshold-like concentration effect, whereby proton availability becomes sufficient at 4 M HCl to substantially destabilize the alkali-roasted matrix and sustain efficient acid penetration through the residual porous layer.

The difference in Nd extraction between 3 and 4 M HCl was further assessed using the replicate measurements conducted under both conditions. The observed divergence—exceeding 50 percentage points—is substantially larger than the experimental variability (RSD < 5%, absolute SD ≤ 2.5 percentage points), confirming that the concentration-dependent increase in extraction is statistically meaningful and cannot be attributed to random experimental variation. In summary, the two-stage concentration study presented here fulfills two distinct scientific objectives: the screening stage establishes the practical operational window (2–6 M), while the kinetic threshold comparison quantifies the mechanistic consequence of operating below the optimum, providing a rigorous justification for the selection of 4 M HCl as the process-defining parameter.

#### 3.2.4 Effect of Roasting Temperature on REE Leaching Efficiency

The conversion of REE-bearing samples into acid-susceptible forms is intrinsically dependent on the efficiency of the preceding alkali roasting reaction. The influence of roasting temperature on the subsequent REE leaching efficiency was systematically evaluated at 500 °C, 600 °C, and 700 °C (Sets D-1, D-2, and D-3, respectively). The variation in elemental extraction efficiency as a function of the thermal decomposition temperature is illustrated in Figure 7.

Roasting conducted at 500 °C resulted in a neodymium (Nd) extraction efficiency of only 42.8%, with lanthanum (La) and cerium (Ce) yielding 24.2% and 25.0%, respectively, indicating that this temperature is insufficient to achieve adequate structural activation of the REE-bearing matrix for subsequent acid leaching. Increasing the temperature to 600 °C provided the optimal condition, leading to a marked improvement in extraction efficiencies across all target elements: Nd reached 74.8%, La reached 42.5%, and Ce reached 40.0%. However, further elevation to 700 °C triggered a sharp decline across all elements, with Nd falling to 32.0%, La to 18.3%, and Ce to 15.0%. Consistent with similar alkali roasting studies in the literature, this loss in extraction efficiency is interpreted as being associated with severe interparticle sintering, agglomeration, and partial glassification occurring at excessive temperatures.^10,32^ These thermal effects are expected to reduce the porosity and reactive surface area of the calcined cake, thereby obstructing the diffusion of the lixiviant acid into the matrix during the subsequent leaching stage. Consequently, an alkali roasting temperature of 600 °C was identified as the most effective condition under the investigated parameters. The observed optimum at 600 °C is further supported by FTIR spectroscopic analysis of the roasted calcines at 500, 600, and 700 °C (Figure 13). At 500 °C, the spectrum exhibits weak and poorly defined absorbance bands (maximum absorbance ∼0.09), with only a broad Fe–O stretching band at ∼630 cm^–1^ and a low-intensity CO_3_^2–^ band at ∼1432 cm^–1^, indicating that phase transformation of the matrix is largely incomplete at this temperature. This is entirely consistent with the low Nd extraction efficiency of 42.8% observed at 500 °C. At 600 °C, the FTIR spectrum undergoes a dramatic change, with absorbance intensities increasing approximately 3-fold relative to the 500 °C calcine. The most prominent feature is a strong band at 789 cm^–1^, which is consistent with the P–O stretching vibration of an orthophosphate or sodium phosphate phase (NaPO_4_/Na_3_PO_4_) formed by the alkali-mediated structural activation of the REE-bearing phosphate matrix during roasting.^32,44^ The concurrent appearance of bands at 727 cm^–1^ (Na-containing phases), 635 cm^–1^ (Fe–O, hematite), and 876 cm^–1^ (CO_3_^2–^ overtone) suggests that 600 °C achieves effective structural activation of the matrix, converting the phosphate framework into acid-susceptible hydroxide and sodium phosphate products. The O–H stretching band at 3574 cm^–1^ provides additional spectroscopic support for the formation of REE hydroxide species. These spectroscopic features collectively support the interpretation that 600 °C represents the effective onset of alkali-mediated phase transformation. At 700 °C, the FTIR spectrum shows a notable shift and broadening of the phosphate-related bands (784 and 717 cm^–1^) relative to the 600 °C pattern, accompanied by the emergence of a new band at 836 cm^–1^. This band is consistent with the formation of sodium iron oxide (NaFeO_2_), a secondary refractory phase known to encapsulate REE-bearing domains and reduce their accessibility to the leaching solution.^32^ The co-occurrence of band shifting and broadening and the emergence of the 836 cm^–1^ feature is consistent with sintering, interparticle consolidation, and the encapsulation of REE phases at excessive roasting temperatures. These observations provide spectroscopic support for the mechanism underlying the sharp decline in Nd extraction efficiency from 74.8% (600 °C) to 32.0% (700 °C). The FTIR findings are further supported by XRD analysis of the roasted calcines (Figure 14a). At 500 °C, the diffractogram is dominated by broad, low-intensity features inherited from the original mineral matrix, with no clearly resolved new crystalline phases, consistent with incomplete phase transformation and the low Nd extraction efficiency of 42.8%. At 600 °C, two new diffraction peaks emerge prominently at 2θ = 16.4° and 34.1°, consistent with the formation of Na_3_PO_4_ and/or NaAlO_2_—sodium-bearing phases expected from the alkali-mediated decomposition of the phosphate and aluminosilicate matrix during roasting. The concurrent appearance of these peaks provides crystallographic support for the structural activation observed in the FTIR spectra and is consistent with the marked improvement in REE extraction efficiency at this temperature. At 700 °C, the diffraction pattern reveals intensification of the 16.5° and 32.8° reflections, consistent with the formation and growth of NaFeO_2_, a thermally stable phase known to encapsulate REE-bearing domains and render them resistant to acid attack.^32,44^ This crystallographic support, combined with the FTIR data, provides a mechanistic basis for the observed temperature-dependent extraction behavior and is consistent with 600 °C being the optimal roasting temperature.

#### 3.2.5 Effect of NaOH Dosage on REE Leaching Efficiency

To determine the optimal reagent stoichiometry for the roasting process, experiments were conducted using 3.75, 7.50, and 11.25 g of sodium hydroxide (NaOH) for a constant 5 g sample mass (sets B-1, D-2, and B-3, respectively). These dosages correspond to ore-to-NaOH mass ratios of 1:0.75, 1:1.50, and 1:2.25. The influence of the alkali-to-ore ratio on the subsequent REE extraction efficiency is illustrated in Figure 8.

Analysis of the experimental data indicates that the leaching efficiencies increase proportionally with the dosage of NaOH incorporated into the roasting medium. At a low alkali-to-ore ratio (1:0.75, set B-1), the Nd extraction efficiency was 53.4%, which improved significantly to 74.9% as the ratio was increased to 1:1.50 (set D-2). Further increasing the NaOH dosage to 11.25 g (1:2.25 ratio, set B-3) yielded the maximum Nd extraction efficiency of 85.4%. While a high NaOH concentration enhances the chemical driving force for decomposition of the monazite matrix, excessive alkali utilization introduces critical operational constraints.

As established in the literature, excessive alkalinity aggressively attacks silicate gangue minerals—such as zircon, feldspar, and quartz—leading to the formation of water-soluble sodium silicate (Na_2_SiO_3_) compounds.^32^ During the subsequent washing and acid leaching stages, these compounds induce polymeric silicate gelation, which elevates the solution viscosity and renders solid–liquid separation (filtration) challenging. Furthermore, high-stoichiometry NaOH consumption necessitates excessive acid for neutralization, thereby escalating industrial reagent costs.^46^ Considering these thermodynamic and physical trade-offs, a roasting temperature of 600 °C with 7.50 g of NaOH (1:1.50 ratio) was identified as the optimal condition to ensure reasonable extraction efficiency while mitigating the risks of gelation and reagent wastage.

### 3.3 Kinetic Modeling and Reaction Mechanism

To elucidate the rate-controlling step, apparent activation energy (*E*_a_), and temperature dependence of the reductive hydrochloric acid leaching process, the dissolution kinetics of REEs—primarily neodymium (Nd), lanthanum (La), and cerium (Ce)—were systematically investigated. The kinetic modeling was primarily established based on neodymium (Nd) to represent the general thermodynamic behavior of the system, given its highest dissolution efficiency. Conversely, the kinetic behavior of cerium (Ce) was evaluated independently due to its distinctive redox-controlled dissolution/precipitation mechanism. Kinetic experiments were performed under optimized chemical conditions (4 M HCl + H_2_O_2_) across four distinct temperatures (60, 70, 80, and 90 °C). Elemental concentrations were monitored by collecting solution samples at predetermined time intervals ranging from 5 to 120 min.

#### 3.3.1 Effect of Temperature on Leaching Kinetics and Total REE Dissolution

An investigation into the synergistic effects of reaction temperature and duration on REE extraction efficiency reveals that elevated temperatures directly stimulate the initial dissolution rates and facilitate mass transfer within the mineral matrix (Figure 9). As the leaching temperature was increased from 60 to 90 °C, the dissolution kinetics significantly accelerated, with the total REE extraction efficiency reaching its peak at the 120 min mark.^24,44^ Notably, neodymium (Nd) achieved a remarkable extraction efficiency of 90.7% at 90 °C (Set K-4), thereby supporting the efficacy of the reductive acid leaching process under optimized conditions.

Despite this overall success in total REE extraction, a characteristic elemental divergence was observed; specifically, the cerium (Ce) extraction efficiency reached an early plateau at 50.0% under identical experimental conditions. Rather than representing a technical limitation, this phenomenon is identified as a natural “selective separation” advantage inherent to the integrated alkali roasting–reductive leaching route. During the high-temperature roasting phase, cerium reacts with atmospheric oxygen and undergoes oxidation to the tetravalent cerianite (CeO_2_) phase, which is exceptionally refractory to standard acidic attack. Due to this thermodynamic anomaly, high-value elements such as Nd and La are efficiently solubilized (>90%), while approximately half of the Ce remains sequestered in the solid residue. This selective retention may reduce the reagent burden and total operational costs associated with subsequent purification stages, such as solvent extraction.^23,46^

#### 3.3.2 Kinetic Modeling and Determination of the Rate-Limiting Step

To elucidate the dissolution mechanism of REEs from the alkali-roasted calcine, the experimental kinetic data for neodymium (Nd) were subjected to a comparative analysis using the Shrinking Core Model (SCM). The mathematical fitness of the data was evaluated against two primary rate-controlling mechanisms: surface chemical reaction control (eq 2) and diffusion control through the porous product layer (eq 3). The apparent rate constants (k) were derived from the slopes of the linear plots, and the precision of the model fit was assessed based on the correlation coefficients (*R* ^2^). The evaluated kinetic parameters across the tested temperature range (60–90 °C) are systematically summarized in Table 5.

**Table 5:** Apparent Rate Constants (*k*_C_, *k*_D_) and Correlation Coefficients (*R* ^2^) for Neodymium (Nd) Dissolution under Different Leaching Conditions

Condition (Temp/Acid/H_2_O_2_)	*k*_C_ (min^–1^)	*R* ^2^ (Chemical Control)	*k*_D_ (min^–1^)	*R* ^2^ (Diffusion Control)
K-1 (60 °C)	0.002241	0.9513	0.000412	0.9513
K-2 (70 °C)	0.003362	0.9468	0.000854	0.9468
K-3 (80 °C)	0.004281	0.9577	0.001258	0.9577
K-4 (90 °C)	0.004835	0.9444	0.001488	0.9493

As presented in Table 5, both the surface chemical reaction and product layer diffusion models provide statistically comparable fits to the experimental data (*R* ^2^ ≈ 0.944–0.958) across the investigated temperature range, indicating that neither mechanism can be exclusively identified as rate-limiting on the basis of regression analysis alone. Although the apparent rate constants obtained from the surface reaction model (*k*_C_) are consistently 3–5 times higher than those derived from the diffusion model (*k*_D_), these parameters arise from different mathematical expressions within the shrinking-core framework and are therefore not directly comparable in absolute magnitude. The similar goodness-of-fit values thus preclude definitive model discrimination.

The Arrhenius analysis further refines the mechanistic interpretation, yielding apparent activation energies of 25.79 kJ/mol (surface reaction model) and 42.95 kJ/mol (diffusion model), as illustrated in Figure 10. Diffusion-controlled systems are typically characterized by relatively low activation energies (<20–25 kJ/mol), whereas chemically controlled reactions exhibit stronger temperature dependence and higher activation barriers (>40 kJ/mol). In the present case, the calculated values lie within the transition region between these classical regimes. The moderate activation energy associated with the surface reaction model suggests that alkali roasting at 600 °C effectively destabilized the refractory phosphate matrix, lowering the intrinsic chemical barrier for dissolution. Conversely, the comparatively high activation energy derived from the diffusion model exceeds the typical range expected for purely diffusion-controlled systems, indicating that internal mass transfer resistance alone cannot adequately describe the rate-limiting step.

Collectively, the comparable statistical fits, the systematic *k*_C_/*k*_D_ disparity, and the intermediate activation energy values support a surface reaction-dominant mixed-control mechanism. Under these conditions, structural modification during roasting facilitates a rapid surface reaction, while proton diffusion and dissolved species transport through the porous product layer continue to impose a measurable kinetic constraint on the overall leaching rate. This behavior is consistent with kinetic trends commonly observed in thermally pretreated secondary rare earth resources. The physical origin of the product layer merits explicit consideration. During alkali roasting at 600 °C, the structural activation of the REE-bearing matrix is interpreted as yielding a mixture of rare earth hydroxides (REE­(OH)_3_), iron oxide phases (predominantly Fe_2_O_3_), and residual aluminosilicate gangue, consistent with the leaching behavior observed. As the acid front progressively dissolves the REE hydroxide phase from the outer surface inward, the insoluble iron oxide skeleton—which accounts for approximately 23.2 wt % of the original matrix—remains as a partially intact porous framework around the dissolving core. This iron-rich residual layer constitutes the principal diffusion barrier, consistent with the elevated activation energy (42.95 kJ/mol) derived from the product layer diffusion model. The rapid and near-complete dissolution of Fe (95.5%) observed under optimized conditions (90 °C, 120 min) indicates that this barrier is eventually disrupted at higher temperatures, which accounts for the strong temperature dependence of the overall extraction rate and the progressive shift toward surface-reaction dominance as temperature increases from 60 to 90 °C. The temporal evolution of this product layer provides additional mechanistic insight. During the initial leaching stage, the acid front rapidly attacks the outer REE-hydroxide-bearing surface, leaving behind a fragmented iron-rich residual framework. As leaching proceeds, this framework itself becomes progressively destabilized and dissolved, as evidenced by the high Fe extraction observed under optimized conditions. Consequently, the diffusion barrier does not simply thicken with time in the manner of classical product-layer systems but may partially self-relax as the iron-rich skeleton is consumed. This provides a plausible explanation for why the extraction curves do not exhibit the sharp plateau typical of strongly diffusion-limited systems and instead remain consistent with a mixed-control regime throughout the leaching period. The pronounced threshold effect observed between 3 and 4 M HCl—wherein Nd extraction increased from approximately 29 to 89% at 80 °C—can be rationalized in the context of the mixed-control mechanism identified in this study. At 3 M HCl, proton availability appears insufficient to simultaneously (i) sustain rapid dissolution of the REE hydroxide surface layer and (ii) maintain adequate H^+^ concentration within the pores of the residual iron-rich product layer. Under these conditions, the diffusion resistance term becomes amplified, and the overall rate becomes only weakly responsive to temperature. This interpretation is consistent with the observation that increasing the temperature from 70 to 80 °C at 3 M HCl yielded only a marginal improvement in Nd extraction (∼3 percentage points), whereas the same temperature increment at 4 M HCl produced a substantially larger gain (∼10 percentage points). Although full kinetic curves were not generated for the 3 M HCl condition, the end point data (27.4% at 70 °C and 30.8% at 80 °C) are consistent with a substantially lower effective dissolution rate, and the weak sensitivity of extraction to temperature at this concentration further supports the interpretation that proton limitation amplifies the diffusion resistance term, thereby suppressing the influence of intrinsic surface-reaction kinetics. The results therefore suggest the existence of a threshold proton activity, above which both the surface reaction and pore-scale acid transport become sufficiently effective to sustain rapid dissolution of the roasted matrix.

### 3.4 Behavior of Impurities and Process Selectivity

Beyond the extraction of rare earth elements, the dissolution behavior of major gangue components—specifically Al, Fe, and Ti—originating from the mineral matrix was systematically analyzed based on the experimental data from the 90 °C trial (Set K-4). The leaching kinetics of these impurities relative to the target REEs are illustrated in Figure 11. This comparative assessment is considered important for evaluating the overall selectivity of the reductive hydrochloric acid leaching process and for estimating the subsequent reagent requirements for downstream purification stages.^30,36^

Aluminum (Al) and iron (Fe) exhibited rapid dissolution throughout the leaching process, reaching extraction efficiencies of 92.9% and 95.5%, respectively, after 120 min. These results indicate that at high temperatures and acid concentrations (4 M HCl), the iron oxide and aluminosilicate structures within the matrix are extensively decomposed.^26,46^ In stark contrast to the other impurities, the titanium (Ti) extraction efficiency remained significantly lower, plateauing at 31.5%.

This phenomenon can be attributed to the thermodynamic instability of solubilized Ti^4+^ ions at elevated temperatures (90 °C), which triggers rapid hydrolysis and subsequent reprecipitation into the solid phase as hydrous titanium dioxide (TiO_2_·*x*H_2_O).^44^ As reported in the literature, this specific behavior of Ti suggests a natural process selectivity against titanium as it may minimize potential issues such as emulsion formation and phase separation complications during subsequent downstream purification (solvent extraction) stages.^16,49^ While the low titanium codissolution is advantageous, the near-complete dissolution of iron (95.5%) and aluminum (92.9%) represents a significant challenge for downstream processing that warrants frank discussion. At the optimal leaching conditions (4 M HCl, 90 °C), the pregnant leach solution (PLS) contains substantial concentrations of Fe^3+^ and Al^3+^ derived from the abundant hematite (23.2 wt %) and aluminosilicate phases (8.54 wt % Al_2_O_3_) in the feedstock. Based on the bulk composition and extraction efficiencies, the molar ratio of [Fe + Al] to [total REE] in the PLS is estimated to be approximately 80:1, indicating that the REE fraction constitutes only a minor component of the total dissolved load. This impurity burden has two principal practical consequences. First, it increases the reagent consumption for any subsequent neutralization or selective precipitation step. Second, high Fe^3+^ concentrations are known to cause crud formation and third-phase splitting during solvent extraction with common acidic extractants (D2EHPA, Cyanex 272), potentially necessitating a selective iron removal step—for instance, by controlled oxidative precipitation as jarosite or goethite—prior to REE separation.^20,48^ It should be noted, however, that the elevated Fe/Al codissolution is an inherent consequence of processing a Fe–Ti-rich heavy mineral matrix under the strongly acidic conditions (4 M HCl) required to achieve high REE extraction. A reduction in acid concentration to 3 M HCl substantially lowered REE extraction to ∼29–30% without meaningfully reducing Fe or Al dissolution, confirming that selective REE leaching cannot be achieved through acid concentration adjustment alone. Future process development should therefore investigate targeted preleach iron removal strategies—such as reductive magnetic separation of hematite—or selective REE stripping circuits to manage this impurity load at the pilot scale.

### 3.5 Effect of Microwave-Assisted Processing and Energy Efficiency

To overcome the limitations of conventional alkali roasting processes—namely, long processing times and high energy consumption—microwave (MW)-assisted roasting experiments (DM-1 and DM-2) were conducted as an innovative process intensification approach. In the microwave experiments, the sample–NaOH mixture was treated for a total of 30 min under a programed heating schedule consisting of a 15 min ramp stage followed by a 15 min holding stage. During treatment, microwave power was automatically modulated by the instrument to maintain the preset roasting conditions. The resulting leaching efficiencies were compared against conventional furnace roasting at 600 °C (D-2) and are presented in Figure 12.

As illustrated in Figure 12, microwave-assisted roasting (set DM-1) enhanced Nd and La extraction efficiencies by +5.3 and +6.1 percentage points, respectively, compared to conventional roasting (set D-2). Under microwave irradiation, Nd and La extraction efficiencies reached 80.1% and 48.6%, whereas conventional roasting yielded 74.8% and 42.5%, respectively. This performance enhancement may be associated with the more rapid and volumetric nature of microwave-assisted heating, which can promote structural modification and improve accessibility of reactive sites during the subsequent leaching stage. Accordingly, the observed increase in Nd and La extraction is interpreted as being consistent with improved structural activation under the applied microwave treatment.

Notably, Ce extraction efficiency exhibited a slight decrease from 40.0% (D-2) to 35.0% (DM-1), suggesting that microwave-assisted thermal treatment may have promoted further oxidation of Ce-bearing species. Regarding impurity behavior, Fe codissolution decreased from 95.1% to 80.0% under microwave conditions, while Ti extraction remained at approximately 15.0%, consistent with the inherent selectivity of the process against titanium. The divergent behavior of Ce relative to Nd and La under microwave-assisted treatment may be attributed to the higher redox sensitivity of cerium during rapid thermal transients. While microwave-assisted heating appears to enhance the structural activation of the roasted matrix and thereby improve Nd and La extraction, Ce-bearing species are more susceptible to further oxidation under accelerated thermal conditions owing to the thermodynamic favorability of the Ce^3+^ → Ce^4+^ transition at elevated temperatures in the presence of atmospheric oxygen. As a result, the modest decrease in Ce extraction observed after microwave treatment is consistent with a competing oxidative passivation effect superimposed on the general enhancement in matrix reactivity. Under this interpretation, microwave-assisted treatment simultaneously improves access to trivalent REE-bearing phases while slightly favoring the persistence of less soluble Ce­(IV)-bearing species—an effect that is effectively reversed by the subsequent addition of H_2_O_2_ as a reductant.

The critical role of H_2_O_2_ in microwave-assisted systems was validated through the DM-2 control experiment (microwave roasting without a reductant). In the absence of H_2_O_2_, the Nd extraction efficiency stabilized at 74.8%, while the Ce extraction declined to approximately 10.0%. This finding indicates that even under microwave irradiation, Ce is rapidly oxidized to the refractory CeO_2_ phase; thus, the addition of a reducing agent remains an essential requirement for achieving meaningful cerium extraction. Collectively, while conventional roasting necessitates prolonged high-temperature processing over several hours, the microwave-assisted route achieves enhanced lanthanide extraction within only 30 min. This substantial reduction in processing time, coupled with improved REE extraction performance, suggests that microwave-assisted roasting may represent a promising direction for energy-conscious processing, warranting further evaluation on larger scales.

To ensure a fair comparison between microwave-assisted and conventional roasting, the microwave treatment was applied under a controlled and reproducible programed schedule. However, bulk thermal equivalence alone does not exclude the possibility of localized heating effects inherent to microwave irradiation. Therefore, the improved extraction observed after microwave treatment is interpreted in terms of enhanced process intensification rather than as a purely temperature-driven effect. XRD analysis of the microwave-roasted calcine (DM-1) provides further mechanistic insight into this behavior (Figure 14b). The diffractogram of the microwave-roasted product is dominated by garnet-group reflections (2θ = 34.7°, 43.9°, 59.6°), which are characteristic of the original mineral matrix, and shows only weak development of the Na-phase peaks (16.4°, 34.1°) that are prominently developed in the conventionally roasted 600 °C calcine. This indicates that bulk phase transformation of the matrix is less complete in the microwave-roasted product within the 30 min treatment time. Paradoxically, however, the microwave-roasted sample yields higher Nd and La extraction efficiencies than the conventionally roasted counterpart. This apparent inconsistency can be rationalized by invoking preferential surface and grain-boundary activation under microwave irradiation: even if the bulk matrix retains much of its original crystalline character, localized structural modification at reactive surfaces and grain boundaries—promoted by the volumetric and rapid nature of microwave heating—may be sufficient to substantially enhance the accessibility of REE-bearing domains to the acid during leaching. This interpretation is consistent with the “surface activation without bulk transformation” concept reported in microwave-assisted mineral processing studies^9,46^ and provides a plausible mechanistic basis for the enhanced extraction performance observed despite the less complete macroscopic phase transformation. Critically, direct microanalytical evidence for this surface activation mechanism is provided by SEM–EDS point analysis of the raw feedstock and the microwave-roasted calcine (Figure 15). EDS analysis of the raw black sand (BM sample) did not detect any REE signal (Ce, La, Nd) above the analytical detection limit, consistent with the submicron grain size and metamict structural state of the monazite-group phase dispersing the REE signal below detectable levels at the analytical scale employed. In contrast, EDS point analysis of the microwave-roasted calcine (DM-1) detected Ce (34.5 wt %), Nd (5.0 wt %), La (4.6 wt %), and Th (7.0 wt %) simultaneously within a single microdomain (Spectrum 52), providing direct microanalytical support for the presence of an REE-bearing phase, consistent with the inferred monazite-group host. This REE-rich microdomain was not detectable in the raw feedstock, strongly suggesting that microwave-assisted roasting selectively exposes and concentrates REE-bearing domains at the particle surface. Collectively, the XRD and SEM–EDS evidence supports the interpretation that microwave-assisted roasting achieves preferential surface-level structural activation of the REE-bearing phase—rendering it accessible to acid attack—without requiring complete bulk phase transformation of the garnet-dominated matrix.

**Figure 15:**
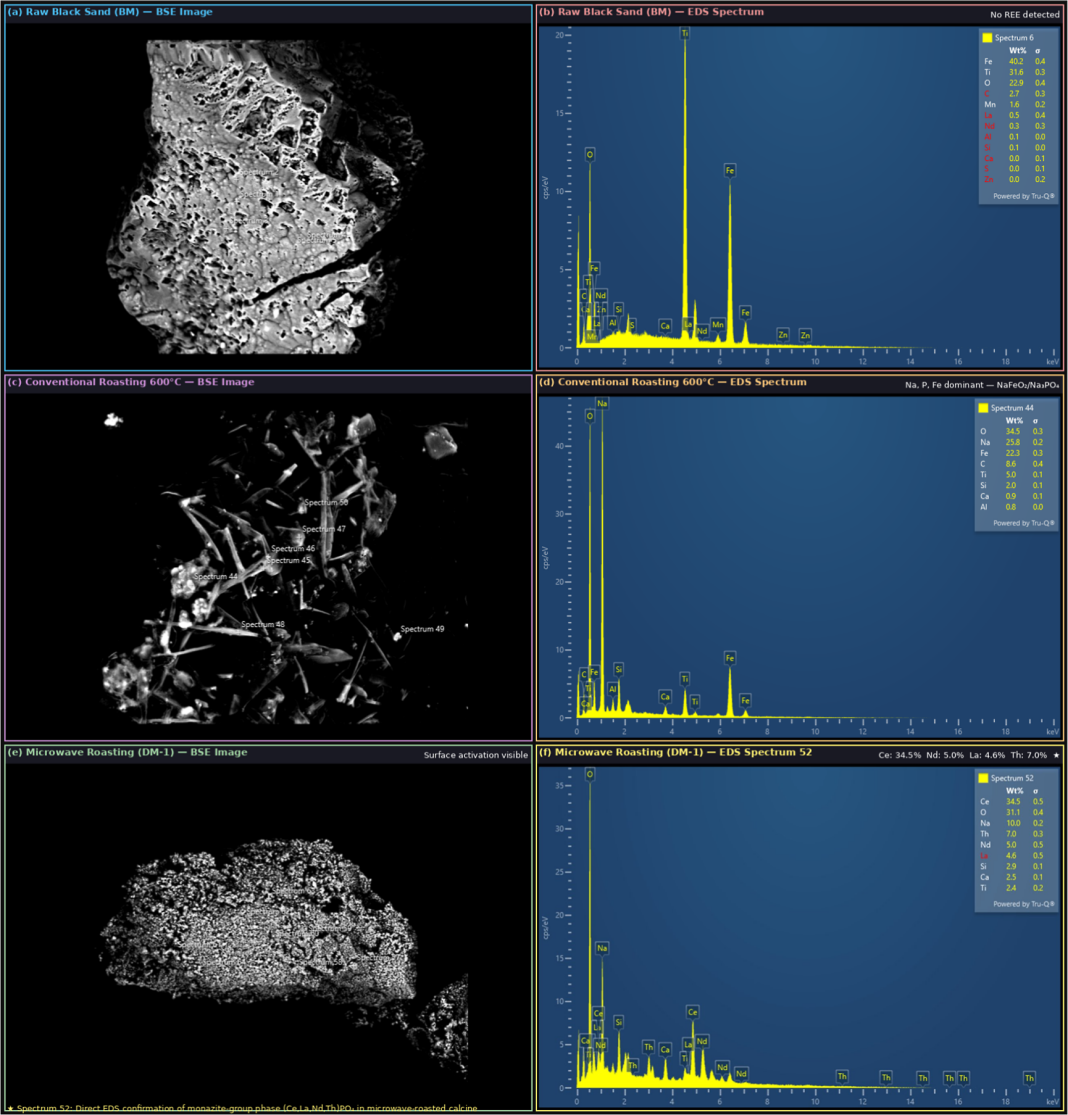
SEM–EDS analysis of the raw black sand feedstock (BM), conventionally roasted calcine (600 °C, 2 h), and microwave-roasted calcine (DM-1, 30 min). (a) BSE image of the raw black sand showing the heterogeneous heavy mineral assemblage. (b) EDS spectrum of the raw black sand: no REE signals detected, consistent with the submicron grain size and metamict structural state of the monazite-group phase. (c) BSE image of the conventionally roasted 600 °C calcine showing modified surface morphology following alkali treatment. (d) EDS spectrum of the 600 °C calcine: Na and P signals prominent, consistent with the formation of sodium phosphate phases, confirmed by XRD and FTIR. (e) BSE image of the microwave-roasted calcine (DM-1) showing a heterogeneous surface texture consistent with localized grain-boundary disruption. (f) EDS point spectrum (Spectrum 52) of the microwave-roasted calcine: Ce (34.5 wt %), Nd (5.0 wt %), La (4.6 wt %), and Th (7.0 wt %) detected simultaneously within a single REE-rich microdomain, providing direct microanalytical evidence for an REE-bearing phase consistent with the inferred monazite-group host [(Ce,La,Nd,Th)­PO_4_]. The contrast between panels (b,f) supports the “surface activation without bulk transformation” mechanism proposed for the enhanced REE extraction performance of the microwave-roasted product.

Collectively, while conventional roasting requires a prolonged furnace treatment, the microwave-assisted route achieved comparable or improved lanthanide extraction within a total treatment time of 30 min. This reduction in processing time suggests a potentially advantageous route in terms of process intensification, although a rigorous specific energy consumption analysis would be required for a quantitative comparison.

### 3.6 Comparative Evaluation with Previous Literature Studies

The efficiency of the integrated alkali roasting–reductive acid leaching process developed in this study was benchmarked against various hydrometallurgical routes reported in the literature for monazite and heavy mineral concentrates. As summarized in Table 6, the current process offers notable advantages in terms of selectivity and reaction kinetics.

**Table 6:** Comparative Performance of the Current Process with the Literature for REE Extraction

Reference	Material	Process Route	Temp./Time	Nd Rec. (%)	Ce Rec. (%)	*E*_a_ (kJ/mol)	Key Advantage
This Work	Black Sand	Alkali Roasting + Reductive Acid Leaching	90 °C/120 min	90.7	50.0	25.8–43.0	High Nd/Ce Selectivity
Abdellah et al.^49^	Pegmatite	Alkali Fusion + Chloride Leaching	80 °C/120 min	92.4	88.6	2.34	High Zr/REE Recovery
Zhang et al.^44^	Monazite	NaOH Roasting + HCl Leaching	90 °C/180 min	88	85	25.4	Standard Alkali Route
Borra et al.^50^	Bauxite Residue	Sulfuric Acid Leaching	90 °C/240 min	75	60	48	Complex Matrix Handling
Zaki^46^	Xenotime	HCl Leaching (Pressure)	150 °C/120 min	94	91	52	High Pressure Efficiency

As evidenced by the comparative data, while many studies aim for maximum dissolution of all REEs, the current method selectively limits cerium extraction to approximately 50% while maintaining high neodymium extraction efficiency (>90%). This selective behavior, coupled with the reduced processing time provided by microwave assistance (30 min vs several hours), distinguishes this work from conventional alkali fusion methods.^44,49^ Furthermore, the activation energy obtained in this study suggests a transition toward a chemically controlled or mixed-controlled mechanism, providing higher sensitivity to temperature changes compared to purely diffusion-controlled systems such as those reported by Abdellah et al.^49^

## 4 Conclusion

This study presents a systematic and mechanistically informed investigation of rare earth element (REE) extraction from heavy mineral tailings of the Manisa–Sart placer deposit, in which a monazite-group phosphate phase is identified as the primary REE-bearing phase by SEM–EDS analysis, via an integrated alkali roasting–reductive acid leaching route. The following conclusions can be drawn from the experimental findings:

Roasting temperature was identified as the most critical pretreatment parameter governing the subsequent leaching performance. Among the investigated temperatures, 600 °C provided optimal structural activation of the REE-bearing matrix, yielding a neodymium (Nd) extraction efficiency of 74.8%. Lower temperatures (500 °C) resulted in insufficient phase transformation, while excessive temperatures (700 °C) caused sintering and surface area loss, reducing Nd extraction efficiency to 32.0%.

Hydrochloric acid (4 M HCl) was established as the most effective leaching agent. Sulfuric acid produced markedly inferior extraction efficiencies due to double sulfate precipitation (NaRE­(SO_4_)_2_), which irreversibly sequestered dissolved REEs back into the solid phase. The optimum NaOH-to-ore ratio was determined to be 1:1.5 (w/w), balancing the extraction efficiency against silicate gelation risks associated with excess alkali.

The addition of hydrogen peroxide (H_2_O_2_) was essential for cerium recovery. Without H_2_O_2_, Ce extraction efficiency remained at approximately 10% due to the formation of insoluble CeO_2_ during roasting. H_2_O_2_ addition enhanced Ce extraction efficiency approximately 4-fold by reductively converting Ce^4+^ to the soluble Ce^3+^ form. The selective partial dissolution of Ce under optimized conditions (∼40.0%) constitutes a natural separation advantage, potentially reducing downstream purification costs.

Leaching kinetics were systematically studied over the temperature range of 60–90 °C. Maximum Nd and Ce extraction efficiencies of 90.7% and 50.0%, respectively, were achieved at 90 °C after 120 min. Analysis using the Shrinking Core Model (SCM) yielded comparable correlation coefficients for both the surface chemical reaction (*R* ^2^ ≈ 0.944–0.958) and product layer diffusion control models, precluding exclusive identification of either as the rate-limiting mechanism. Arrhenius analysis yielded apparent activation energies of 25.79 kJ/mol (chemical reaction model) and 42.95 kJ/mol (diffusion model), both falling within the transition region between classical diffusion- and chemically controlled regimes. These results collectively support a surface reaction-dominant mixed-control dissolution mechanism.

Microwave-assisted roasting conducted over a total treatment time of 30 min (15 min ramp +15 min hold) enhanced Nd and La extraction efficiencies relative to conventional furnace roasting (2 h), reaching 80.1% and 48.6%, respectively. SEM–EDS analysis directly confirmed that microwave-assisted roasting exposes REE-bearing phosphate domains at the particle surface—rendering them detectable by EDS and accessible to acid attack—without requiring complete bulk phase transformation. This “surface activation without bulk transformation” mechanism is established by the combined XRD and SEM–EDS evidence and provides a direct mechanistic explanation for the superior extraction performance of the microwave-roasted product. The process selectivity against titanium was observed under all conditions, with Ti dissolution limited to 31.5%, owing to hydrolysis and reprecipitation of Ti^4+^ at elevated temperatures.^38^ Overall, the findings suggest a viable and energy-conscious hydrometallurgical pathway for REE recovery from a previously underexplored Turkish secondary resource, offering mechanistic insights relevant to the broader development of sustainable rare earth supply chains.
